# Continuous Differentiability of the Value Function of Semilinear Parabolic Infinite Time Horizon Optimal Control Problems on $L^2(\Omega )$ Under Control Constraints

**DOI:** 10.1007/s00245-022-09840-9

**Published:** 2022-04-13

**Authors:** Karl Kunisch, Buddhika Priyasad

**Affiliations:** 1 Institute for Mathematics and Scientific Computing, University of Graz, Heinrichstrasse 36, 8010 Graz, Austria; 2 Radon Institute, Austrian Academy of Science, Linz, Austria

**Keywords:** Value Function, Infinite horizon optimal control, Hamilton-Jacobi-Bellman equation, Differentiability, Semi-linear parabolic equation

## Abstract

An abstract framework guaranteeing the local continuous differentiability of the value function associated with optimal stabilization problems subject to abstract semilinear parabolic equations subject to a norm constraint on the controls is established. It guarantees that the value function satisfies the associated Hamilton–Jacobi–Bellman equation in the classical sense. The applicability of the developed framework is demonstrated for specific semilinear parabolic equations.

## Introduction

Continuous differentiability of the value function with respect to the initial datum is an important problem in optimal feedback control theory. Indeed, if the value function is $C^1$ then it is the solution of a Hamilton Jacobi Bellman (HJB) equation and its negative gradient can be used to define on optimal state feedback law. The subject matter of this paper addresses local continuous differentiability of the value function ${\mathcal {V}}$ for infinite horizon optimal control problems subject to semilinear parabolic control problems and norm constraints on the control. Such problems are intimately related to stabilization problems which are often cast as infinite horizon optimal control problems. Investigating infinite horizon problems constitutes one of the specificities of this paper. Another one is the fact that we focus on the differentiability of ${\mathcal {V}}$ on (subsets of) $L^2(\Omega )$. Thus we need to consider the semilinear equations with initial data $y_0 \in L^2(\Omega )$. As a consequence the solutions of the semilinear equations only enjoy low Sobolev-space regularity. This restricts the class of nonlinearities, compared to those which are admissible if the states are in $L^\infty ((0,\infty )\times \Omega )$, which is the situation typically addressed in the literature on optimal control [8] and [25]. The latter necessitates to take the initial conditions in spaces strictly smaller than $L^2(\Omega )$. Here we consider $L^2(\Omega )$, first due to intrinsic interest, secondly because ultimately the HJB equation should be solved numerically, which is easier in an $L^2(\Omega )$ setting than in other topologies, like $H^1(\Omega )$. Let us also recall that one of the approaches to solve the HJB equation is given by the policy iteration. It assumes that the value function is $C^1$.

The underlying analysis demands stability and sensitivity analysis of infinite dimensional optimal control problems subject to nonlinear equations. For this purpose we utilize the theory of generalized equations as established by [10] and [23]. It involves first order approximations of the state and adjoint equations, which lead to restrictions on the class of nonlinearities which can be admitted. We refer to the section on examples in this respect.

The current investigations are to some degree a continuation of work the first author’s work on optimal feedback control for infinite dimensional systems. In [4–6] Taylor approximations of the value function for problems with a concrete structure, namely, bilinear control systems, and the Navier Stokes equations were investigated and differentiability of the value function was obtained as a by product. In these investigations norm constraints were not considered. Here we admit norm constraints and we focus on semilinear equations. Let us also notice that the systems investigated in [4–6] share the property that the second derivatives with respect to the state variable of the nonlinearity in the state equation do not depend on the state itself anymore.

Let us also compare our work to the developments in the field of parametric sensitivity analysis of semilinear parabolic equations under control constraints. There are many papers focusing on stability and sensitivity analysis of finite time horizon problems along with pointwise control constraints, see e.g. [2, 13–15, 18, 19, 26, 27], and the literature there. First, of these papers, except for [14], consider the case with initial data in $L^2(\Omega )$. In [14] again the third derivative of the nonlinearity is zero. Secondly, all of them consider the finite horizon case. Since we treat infinite horizon problems we have to guarantee stabilizability (for small initial data) under control constraints. Then we use a fixed point argument to obtain well-posedness of the system. Well-posedness and stability with respect to parameters of the adjoint equation is significantly more involved for infinite horizon problems than for finite horizon problems. It requires techniques, differently from those used in the finite horizon case. Another aspect is the proper characterization of the adjoint state at $t=\infty$.

In the finite dimensional case, there is, of course a tremendous amount of work on the treatment of the value function if it is not $C^1$. Fewer papers concentrate on the case where the value function enjoys smoothness properties. We mention [12] and [7] in this respect.

In order to achieve the goal we desire, we lay out the following setup. In Sect. 2, we consider an abstract parametric optimization problem with an equality constraint and another convex constraint. Existence of an optimal solution, of a multiplier associate to the equality constraint, and Lipschitz stability of the component of the state variable which lies in the complement of the kernel of the linearized constraint will be established. This result is necessary but not sufficient for the further developments, since stability is obtained in a norm which is too weak and since the stability estimate does not involve the component in the kernel of the linearized constraint and the multiplier, i.e. the adjoint states, yet. At the level of Sect. 2 this remains as Assumption (H7). In Sect. 3 we specify the concrete optimal stabilization problem and a set of conditions, most importantly on the nonlinearity of the state equation, under which Assumption (H7) can be established, for initial data $y_0 \in L^2(\Omega )$. Section 3 also contains a summary of the main results of this paper. They are stated as theorems with a little stronger assumptions than eventually necessary, for the saker of easing the presentation. Section 4 is dedicated to the proof of verifying the assumptions of the general setup of Sect. 2 for the concrete optimal control problem stated in Sect. 3. As conclusion we obtain the Lipschitz continuity in the appropriate norms of the all variables appearing in the optimality system with respect to the parameter of interest, which is the initial condition $y_0$, in our case. Since our analysis is a local one involving second order optimality conditions, solutions to the optimality system are related to local solutions to the optimal control problem. As a corollary to these results we obtain that the local value function is Fréchet differentiable. In Sect. 5, we show that in the neighborhood of global solutions the value function ${\mathcal {V}}$ satisfies the Hamilton–Jacobi–Bellman (HJB) equation in the strong sense. Finally, Sect. 6 is devoted to demonstrating that the developed framework is applicable for some concrete examples, namely for linear systems, Fisher’s equations, and parabolic equations with global Lipschitz nonlinearities. All our results require a smallness assumption on the initial conditions $y_0$. Two aspects need to be taken into consideration in this respect. First $y_0$ has to be sufficiently small so that the controlled system is stable. Secondly a second order optimality condition is needed. For this to hold a sufficient condition is provided by smallness of the adjoint state, which in turn can be implied by smallness of $y_0$. We stress that these two issues are of related, but independent nature.

## Lipschitz Stability for an Abstract Optimization Problem

Here we present a stability result for an abstract, infinite dimensional optimization problem which will be the building block for the results below. This result is geared towards exploiting the specific nature of optimization problem with differential equations as constraints. First existence of a dual variable will result from a regular point condition. Subsequently the Lipschitz stability result is obtained in two steps. In the first one, we rely on the relationship between the linearized optimality conditions and an associated linear-quadratic optimal optimization problem, with an extra convex constraint. This approach is useful since it provides the existence of solutions to the linearized system on the basis of variational techniques. However it dictates a certain norms for the involved quantities. These norms are too weak for our goal of obtaining Lipschitz continuity of the adjoint variables in such a manner that differentiability of the cost with respect to the initial conditions can be argued. Therefore, in a second step we exploit the specific structure of the optimality systems, using the fact that it is related to a parabolic optimal control problem, to obtain the Lipschitz continuity in the stronger norms. This two step approach is also present in some of the earlier work on stability and sensitivity analysis which was quoted in the introduction. But due to that fact these papers considered finite horizon problems it came as a byproduct which improved the regularity of the adjoints. In our work it is essential to reach our goal. This is why we decided to formalize this two step approach which was not done in earlier work.

Concretely, we consider the optimization problem 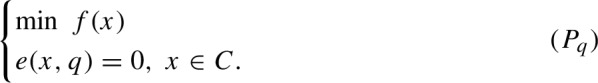 with a parameter dependent equality constraint, and a general constraint described by $x \in C$, where *C* is a closed convex subset of a real Hilbert space *X*. Further, *W* is a real Hilbert space and *P* is a normed linear space. In the application that we have in mind, the parameter *q* will appear as the initial condition in the dynamical system. The following Assumption (H1) is assumed to hold throughout.

### Assumption H1

$q_0 \in P$ is a nominal reference parameter,

$x_0$ is a local solution ($P_{q_0}$),

$f: X \longrightarrow {\mathbb {R}}^+$ is twice continuously differentiable in a neighborhood of $x_0$,

$e: X \times P \longrightarrow W$ is continuous, and twice continuously differentiable w.r.t. *x*, with first and second derivative Lipschitz continuous in a neighborhood of $(x_0,q_0)$.

The derivatives with respect to *x* will be denoted by primes and the derivatives w.r.t. *y* and *u* later on, are denoted by subscripts. They are all considered in the sense of Lebesgue derivatives.

We introduce the Lagrangian ${{\mathcal {L}}}: X \times P \times W^* \longrightarrow {\mathbb {R}}$ associated to ($P_q$) by

2.1 $$\begin{aligned} {{\mathcal {L}}}(x, q, \lambda ) = f(x) + \langle \lambda , e(x,q) \rangle _{W^*, W}. \end{aligned}$$

Next further relevant assumptions are introduced:

### Assumption H2

(regular point condition)

$$\begin{aligned} 0 \in \text {int } e'(x_0, q_0)(C - x_0), \end{aligned}$$

where *int* denotes the interior in the *W* topology. This regularity condition implies the existence of a Lagrange multiplier $\lambda _0 \in W^*$, see e.g. [20] such that the following first order condition holds:

2.2 $$\begin{aligned} {\left\{ \begin{array}{ll} \langle {{\mathcal {L}}}'(x_0, q_0, \lambda _0), c - x_0 \rangle _{X^*,X} \ge 0, \ \forall c \in C,\\ e(x_0, q_0) = 0. \end{array}\right. } \end{aligned}$$

It is equivalent to

2.3 $$\begin{aligned} {\left\{ \begin{array}{ll} 0 \in {{\mathcal {L}}}'(x_0, q_0, \lambda _0) + \partial \mathbf {I}_C(x_0), \quad &{}\text {in } X^*,\\ e(x_0, q_0) = 0, \quad &{}\text {in } W, \end{array}\right. } \end{aligned}$$

where $\partial \mathbf {I}_C(x)$ denotes the subdifferential of the indicator function of the set *C* at $x \in X$.

Let $\displaystyle A \in {{\mathcal {L}}}(X, X^*)$ denote the operator representation of $\displaystyle {{\mathcal {L}}}''(x_0, q_0, \lambda _0)$, i.e.

2.4 $$\begin{aligned} \langle Ax_1, x_2 \rangle _{X^*,X} = {{\mathcal {L}}}''(x_0, q_0, \lambda _0)(x_1 ,x_2) \end{aligned}$$

and define

2.5 $$\begin{aligned} E = e'(x_0, q_0) \in {{\mathcal {L}}}(X,W). \end{aligned}$$

We further require

### Assumption H3

(positive definiteness)

$$\begin{aligned} \exists \kappa > 0: \ \langle Ax, x \rangle _{X^*, X} \ge \kappa \left\Vert x\right\Vert ^2_X, \ \forall x \in \text {ker } E. \end{aligned}$$

Condition (H3) is a bit stronger than a second order sufficient optimality condition, since it does not take into consideration the activity or inactivity of the constraints. Such weaker second order conditions typically allow to derive quadratic positive definite lower bounds on the cost and Hölder continuity with respect to perturbations. For Lipschitz continuity and differentiability stronger assumptions, such as (H3) are typically assumed. We refer exemplarily to [13–15, 27], and [16, Sect. 2.3]. The constraints in these references, however, are not identical with those of the present paper.

The stability result of $(x_0, \lambda _0)$ with respect to perturbation of *q* at $q_0$ will be based on Robinson’s strong regularity condition which involves the following linearized form of the optimality condition,

2.6 $$\begin{aligned} {\left\{ \begin{array}{ll} 0 \in {{\mathcal {L}}}'(x_0, q_0, \lambda _0) + A(x - x_0) + E^*(\lambda - \lambda _0) + \partial \mathbf {I}_C(x) &{}\text {in } X^*,\\ 0 = e(x_0, q_0) + E(x - x_0) &{}\text {in } W. \end{array}\right. } \end{aligned}$$

We define a multivalued operator $\displaystyle {{\mathcal {T}}}: X \times W^* \longrightarrow X^* \times W$ by

2.7 $$\begin{aligned} {{\mathcal {T}}}\begin{pmatrix}x \\ \lambda \end{pmatrix}= \begin{pmatrix}A &{} E^* \\ E &{} 0 \end{pmatrix}\begin{pmatrix}x \\ \lambda \end{pmatrix}+ \begin{pmatrix}f'(x_0) - A x_0 \\ -E x_0 \end{pmatrix}+ \begin{pmatrix}\partial \mathbf {I}_C(x) \\ 0 \end{pmatrix}, \end{aligned}$$

and observe that (2.6) is equivalent to

$$\begin{aligned} \displaystyle 0 \in {{\mathcal {T}}}\begin{pmatrix}x \\ \lambda \end{pmatrix}. \end{aligned}$$

Here it is understood that ${{\mathcal {T}}}$ is evaluated at $(x_0,q_0,\lambda _0)\in X\times P\times W^*$. But ${{\mathcal {T}}}$ is not yet the mapping for which we need to verify the Robinson–Dontchev strong regularity condition in our context. It relates to the fact that we must to treat the multiplier $\lambda$ in smaller space than $W^*$. Before we can properly specify this condition some additional preparation is necessary. We first introduce Banach spaces:

2.8 $$\begin{aligned} \underline{X} \subset X, \ \underline{W^*} \subset W^*, \ \underline{X^*} \subset X^*, \end{aligned}$$

with continuous injections. We emphasize that $\underline{X^*}$ should not be confused with $(\underline{X})^*$. A restriction of ${{\mathcal {T}}}$ will be defined as multivalued operator $\displaystyle \underline{{{\mathcal {T}}}}: \underline{X} \times \underline{W^*} \rightarrow \underline{X^*} \times W$. Indeed, in applications to optimal control problems extra regularity of multipliers can be obtained by investigating the solutions (2.3), see e.g. Sect. 3. In the context of optimal stabilization problems this structural property will become transparent in Propositions 1 and 2, see also [6, Proposition 15]. It will turn out to be essential for our purposes. But this situation where the multiplier has extra regularity is also of abstract interest. When studying stability in this setting this means that the second coordinate of the domain of ${{\mathcal {T}}}$ needs to be changed from $W^*$ to $\underline{W^*}$. This entails that the range space of ${{\mathcal {T}}}$ has to be modified appropriately, in order to obtain stability of the $\lambda$ coordinate. For this purpose we introduce $\underline{X^*} \subset X^*$. The reason for further restricting *X* to $\underline{X}$ will become evident in the proof of Proposition 2. It is related to the fact that we consider infinite horizon problems. A concrete use of these space is elaborated in detailed in Sect. 3.2.2.

Now we adapt the conditions on *f* and *e* to the choice of the spaces in (2.8).

### Assumption H4

There exists a neighborhood $\displaystyle \widetilde{U}_1 \times \widetilde{U}_2 \subset \underline{X} \times P$ of $(x_0, q_0)$ such that

(i) the restriction of $x \mapsto f'(x)$ to $\underline{X}$ defines a mapping $\underline{f'}(x)$ from $\widetilde{U}_1 \subset \underline{X}$ to $\underline{X^*}$,
(ii) the restriction $e'(x,q)^* \in {{\mathcal {L}}}(W^*,X^*)$ to $\underline{W^*}$ defines operators $\underline{e'(x,q)^*} \in {{\mathcal {L}}}(\underline{W^*},\underline{X^*})$ for every $(x,q) \in \widetilde{U}_1 \times \widetilde{U}_2$.

With these assumption holding we define the restricted linearized Lagrangian

2.9 $$\begin{aligned}&\underline{{{\mathcal {L}}}'}: \widetilde{U}_1 \times \widetilde{U}_2 \times \underline{W^*} \subset \underline{X} \times P \times \underline{W^*} \nonumber \\&\quad \longrightarrow \underline{X^*} \quad \text {by} \quad \underline{{{\mathcal {L}}}'}(x,q,\lambda ) = \underline{f'}(x) + \underline{e'(x,q)^*}\lambda . \end{aligned}$$

Next we adapt $\partial \mathbf {I}_{C} \subset X^*$ to the situation of (2.8) and define for $x \in \underline{X}$ the set valued mapping

2.10 $$\begin{aligned} {\underline{\partial \mathbf {I}_{C}}(x)} = \left\{ y \in \underline{X^*}: \langle y, v - x \rangle _{X^*,X} \le 0, \ \forall v \in C \cap \underline{X} \right\} \subset \underline{X^*}. \end{aligned}$$

We henceforth assume that $(x_0,\lambda _0) \in \underline{X} \times \underline{W^*}$, it will also follow as a special case of (H7). The following assumption will guarantee that the restriction $\underline{{{\mathcal {T}}}}$ of ${{\mathcal {T}}}$ is well-defined as operator from $\underline{X} \times \underline{W^*}$ to $\underline{X^*} \times W$, and the one beyond is needed for Lipschitz continuous dependence of local solutions to ($P_q$) with respect to *q*.

### Assumption H5

$\displaystyle \underline{{{\mathcal {L}}}'}: \widetilde{U}_1 \times \widetilde{U}_2 \times \underline{W^*} \subset \underline{X} \times P \times \underline{W^*} \longrightarrow \underline{X^*}$ is Fréchet differentiable with respect to *x*, and $(\underline{{{\mathcal {L}}}'})'$, as a mapping $(x,q,\lambda ) \mapsto (\underline{{{\mathcal {L}}}'})'(x,q,\lambda )$, is continuous at $(x_0,q_0,\lambda _0) \in \underline{X} \times P \times \underline{W^*}$.

### Assumption H6

There exists $\nu > 0$ such that:

2.11a $$\begin{aligned} \left\Vert e(x,q_1) - e(x,q_2)\right\Vert _W&\le \nu \left\Vert q_1 - q_2\right\Vert _{P}, \forall (x,q_1) \text { and } (x,q_2) \in \widetilde{U}_1 \times \widetilde{U}_2, \end{aligned}$$

2.11b $$\begin{aligned} \left\Vert {\underline{e'(x,q_1)^*} - \underline{e'(x,q_2)^*}} \right\Vert _{{{\mathcal {L}}}(\underline{W^*},\underline{X^*})}&\le \nu \left\Vert q_1 - q_2\right\Vert _{P}, \forall (x,q_1) \text { and } (x,q_2) \in \widetilde{U}_1 \times \widetilde{U}_2. \end{aligned}$$

Let us further set

$$\begin{aligned} \underline{E^*} = \underline{e'(x_0,q_0)^*} \in {{\mathcal {L}}}(\underline{W^*},\underline{X^*}) \text { and } \underline{A} = (\underline{{{\mathcal {L}}}'}(x_0,q_0,\lambda _0))' \in {{\mathcal {L}}}(\underline{X},\underline{X^*}). \end{aligned}$$

With Assumptions (H1)–(H5) holding (2.3) can be expressed as

2.12 $$\begin{aligned} {\left\{ \begin{array}{ll} 0 \in \underline{{{\mathcal {L}}}'}(x_0, q_0, \lambda _0) + \underline{\partial \mathbf {I}_C}(x_0), \quad &{}\text {in } \underline{X^*},\\ e(x_0, q_0) = 0, \quad &{}\text {in } W. \end{array}\right. } \end{aligned}$$

Moreover (2.6) restricted to $\underline{X}\times \underline{X^*}$ result in:

2.13 $$\begin{aligned} 0 \in {\left\{ \begin{array}{ll} \underline{{{\mathcal {L}}}'}(x_0, q_0, \lambda _0) + \underline{A}(x - x_0) + \underline{E^*}(\lambda - \lambda _0) + \underline{\partial \mathbf {I}_C}(x) &{} \text { in } \underline{X^*},\\ e(x_0, q_0) + E(x - x_0) &{} \text { in } W, \end{array}\right. } \end{aligned}$$

and the multivalued operator $\displaystyle \underline{{{\mathcal {T}}}}: \underline{X} \times \underline{W^*} \longrightarrow \underline{X^*} \times W$ related to (2.7) is defined as

2.14 $$\begin{aligned} \underline{{{\mathcal {T}}}} \begin{pmatrix}x \\ \lambda \end{pmatrix}= \begin{pmatrix}\underline{A} &{} \underline{E^*} \\ E &{} 0 \end{pmatrix}\begin{pmatrix}x \\ \lambda \end{pmatrix}+ \begin{pmatrix}\underline{f'}(x_0) - \underline{A} x_0 \\ -E x_0 \end{pmatrix}+ \begin{pmatrix}\underline{\partial \mathbf {I}_C}(x) \\ 0 \end{pmatrix}. \end{aligned}$$

Observe that (2.13) is equivalent to

$$\begin{aligned} \displaystyle 0 \in \underline{{{\mathcal {T}}}} \begin{pmatrix}x \\ \lambda \end{pmatrix}. \end{aligned}$$

Existence and Lipschitz continuity of solutions in a neighborhood $(x_0, q_0, \lambda _0)$ will follow from the strong regularity assumption which requires us to show that there exist neighborhoods ${\hat{V}} \subset \underline{X^*} \times W$ of 0 and ${\hat{U}} = {\hat{U}}_1 \times {\hat{U}}_2 \subset \underline{X} \times \underline{W^*}$ of $(x_0, q_0)$ such that $\underline{{{\mathcal {T}}}}^{-1}$ has the properties that $\underline{{{\mathcal {T}}}}^{-1}({\hat{V}}) \cap {\hat{U}}$ is single-valued and that it is Lipschitz continuous from ${\hat{V}}$ to ${\hat{U}}$, see [10], (and also [23, 16, Definition 2.2, p. 31], in case $\underline{X} =X, \ \underline{W^*} = W^*, \ \underline{X^*} =X^*$). We approach the strong regularity assumption in two steps. In the first one we argue invertibility of ${{\mathcal {T}}}$ and Lipschitz continuity of the variable *x* in *X*. For this purpose we exploit the symmetry of ${{\mathcal {T}}}$ and consider an associated variational problem. In our specific situation the inverse of ${{\mathcal {T}}}$—and consequently of $\underline{{{\mathcal {T}}}}$—is single-valued and thus the restriction to the neighborhood ${{\hat{U}}}$ is not needed. Existence and Lipschitz continuity of $\lambda$ as well as Lipschitz continuity of *x* in the small space $\underline{X}\times \underline{W^*}$ remains an assumption in the generality of problem ($P_q$). It will be verified in a second step for the optimal stabilization problems in the following sections.

### Assumption H7

For $(\beta _1, \beta _2) \in {\hat{V}} \subset \underline{X^*} \times W$ , the solution $\displaystyle \left( x_{(\beta _1,\beta _2)}, \lambda _{(\beta _1,\beta _2)} \right)$ to $$\displaystyle {{\mathcal {T}}}\begin{pmatrix}x \\ \lambda \end{pmatrix}= \begin{pmatrix}\beta _1 \\ \beta _2 \end{pmatrix}$$ lies in $\underline{X} \times \underline{W^*}$. Moreover there exists a constant $k > 0$ such that

$$\begin{aligned}&\left\Vert x_{(\beta _1,\beta _2)} - x_{({\hat{\beta }}_1,{\hat{\beta }}_2)}\right\Vert _{\underline{X}} + \left\Vert \lambda _{(\beta _1,\beta _2)} - \lambda _{({\hat{\beta }}_1,{\hat{\beta }}_2)}\right\Vert _{\underline{W^*}}\\&\quad \le k \left[ \left\Vert (\beta _1,\beta _2) - ({\hat{\beta }}_1,{\hat{\beta }}_2)\right\Vert _{\underline{X^*} \times W} + \left\Vert x_{(\beta _1,\beta _2)} - x_{({\hat{\beta }}_1,{\hat{\beta }}_2)}\right\Vert _X \right] \end{aligned}$$

for all $$(\beta _1,\beta _2) \in {\hat{V}}, ({\hat{\beta }}_1,{\hat{\beta }}_2) \in {\hat{V}}$$.

This condition is used after the existence of $x_\beta =x_{(\beta _1,\beta _2)}$ was already established. Note that for $(\beta _1, \beta _2)^T = 0$ we have $(x_{(0,0)},\lambda _{(0,0)}) = (x_0, \lambda _0)$ and hence (H7) in particular implies that $(x_0, \lambda _0) \in \underline{X} \times \underline{W^*}$.

We arrive at the announced stability result.

### Theorem 2.1

Assume that (H1)–(H7) hold at a local solution $x_0$ of ($P_{q_0}$). Then there exist a neighborhood $U = U(x_0,\lambda _0) \subset \underline{X} \times \underline{W^*}$, a neighborhood $N = N(q_0)\subset P$, and a constant $\mu$ such that for all $q \in N$ there exists a unique $(x(q), \lambda (q)) \in U$ satisfying

2.15 $$\begin{aligned} 0 \in {\left\{ \begin{array}{ll} \underline{{{\mathcal {L}}}'}(x(q), q, \lambda (q)) + \underline{\partial \mathbf {I}_C} (x(q)), \quad &{}\text { in } \underline{X^*}, \\ e(x(q), q), \quad &{}\text { in } W, \end{array}\right. } \end{aligned}$$

and

2.16 $$\begin{aligned} \left\Vert (x(q_1), \lambda (q_1)) - (x(q_2), \lambda (q_2))\right\Vert _{\underline{X} \times \underline{W^*}} \le \mu \left\Vert q_1 - q_2\right\Vert _{P}, \ \forall q_1,q_2 \in N.\qquad \end{aligned}$$

In addition there exists a nontrivial neighborhood $\widetilde{N} \subset N$ of $q_0$ such that *x*(*q*) is a local solution of ($P_q$) for $q \in \widetilde{N}$.

For the proof we shall employ the following lemma in which $A \in {{\mathcal {L}}}(X,X^*)$ and $E \in {{\mathcal {L}}}(X,W)$ denote generic operators. For the sake of completeness we also include its proof.

### Lemma 2.2

Let $({\tilde{a}}, {\tilde{b}}) \in X^* \times W$, assume that $A\in {{\mathcal {L}}}(X,X^*)$ is self-adjoint and satisfies (H3), and that the set $\displaystyle S({\tilde{b}}) = \{ x \in C: \ E x = {\tilde{b}} \}$ is nonempty. Then the problem

2.17 $$\begin{aligned} {\left\{ \begin{array}{ll} \min _{x \in C} {\tilde{J}}(x) = \min _{x \in C} \frac{1}{2}\langle Ax, x \rangle _{X^*,X} + \langle {\tilde{a}}, x \rangle _{X^*,X},\\ E x = {\tilde{b}}, \end{array}\right. } \end{aligned}$$

admits a unique solution $x = x({\tilde{a}}, {\tilde{b}})$ satisfying

2.18 $$\begin{aligned} \langle A x + {\tilde{a}}, v - x \rangle _{X^*,X} \ge 0, \ \text {for all } v \in S({\tilde{b}}). \end{aligned}$$

If moreover the regular point condition $0 \in \text {int } E(C - x({\tilde{a}}, {\tilde{b}}))$ holds, then there exists $\lambda = \lambda ({\tilde{a}}, {\tilde{b}}) \in W^*$ such that

2.19 $$\begin{aligned} 0 \in {\left\{ \begin{array}{ll} \begin{pmatrix}A &{} E^* \\ E &{} 0\end{pmatrix}\begin{pmatrix}x \\ \lambda \end{pmatrix}+ \begin{pmatrix}{\tilde{a}} \\ -{\tilde{b}} \end{pmatrix}+ \begin{pmatrix}\partial \mathbf {I}_C(x) \\ 0 \end{pmatrix}. \end{array}\right. } \end{aligned}$$

### Proof

Since *C* is a closed and convex, $S({\tilde{b}})$ is closed and convex. By assumption $S({\tilde{b}})$ is nonempty. Hence there exists an $x \in C$ such that $Ex = {\tilde{b}}$. Note that each such *x* can be uniquely decomposed as $x = w + y$, with $y \in \text {ker} E,\, w \in \text {ker}E^\perp$ and $E w = {\tilde{b}}$. By (H3) the functional ${\tilde{J}}$ is bounded from below and coercive on $S({\tilde{b}})$. Hence there exists a minimizing sequence $\{ x_n \}$ in $S({\tilde{b}})$ such that $\displaystyle \lim _{n \rightarrow \infty } {\tilde{J}}(x_n) = \inf _{x \in S({\tilde{b}})} {\tilde{J}}(x)$. Each $x_n$ can be decomposed as $x_n = w + y_n$, with $y_n \in \text {ker} E$. By (H3) the sequences $\displaystyle \{y_n\}_{n=1}^\infty$ and hence $\displaystyle \{x_n\}_{n=1}^\infty$ are bounded. Thus there exists a subsequence $\displaystyle \{ x_{n_k} \}$ with weak limit $x=x({\tilde{a}}, {\tilde{b}})$ in $S({\tilde{b}})$. Since ${\tilde{J}}$ weakly lower semi-continuous, we have that $\displaystyle {\tilde{J}}(x) \le \liminf _{k \rightarrow \infty } {\tilde{J}}(x_{n_k})$ and *x* minimizes ${\tilde{J}}$ over $S({\tilde{b}})$. This further implies that $\displaystyle \langle Ax + {\tilde{a}}, v - x \rangle _{X^*,X} \ge 0$ for all $v \in S({\tilde{b}})$. Uniqueness of *x* follows from (H3).

The regular point condition implies the existence of a multiplier $\lambda = \lambda ({\tilde{a}}, {\tilde{b}})\in W^*$ such that (2.19) holds. See e.g. [16, Theorem 1.6] $\square$

### Proof of the Theorem 2.1

(i) The proof of the first assertion of the Theorem 2.1 is based on the implicit function theorem of Dontchev for generalized equations, see [10, Theorem 2.4, Remark 2.5]. We introduce the mapping $\underline{{{\mathcal {F}}}}: \underline{X} \times P \times \underline{W^*} \longrightarrow \underline{X^*} \times W$ given by
  $$\begin{aligned} \underline{{{\mathcal {F}}}}(x,q,\lambda )= \begin{pmatrix}\underline{{{\mathcal {L}}}'}(x,q,\lambda ) \\ e(x,q) \end{pmatrix}, \end{aligned}$$
  and observe that Assumption (H6) implies that for all $(x,q_1,\lambda ),$ and $(x,q_2,\lambda ) \in \widetilde{U}_1 \times \widetilde{U}_2 \times \underline{W^*}$
  2.20 $$\begin{aligned} \left\Vert \underline{{{\mathcal {F}}}}(x,q_1,\lambda ) - \underline{{{\mathcal {F}}}}(x,q_2,\lambda )\right\Vert _{\underline{X^*} \times W} \le \nu \left( 1 + \left\Vert \lambda \right\Vert _{\underline{W^*}} \right) \left\Vert q_1 - q_2\right\Vert _{P}. \end{aligned}$$
  By (H1) and (H5), and using the integral mean value theorem it can be argued that
  $$\begin{aligned} \begin{pmatrix}x \\ \lambda \end{pmatrix}\rightarrow \begin{pmatrix}\underline{A} &{} \underline{E^*} \\ E &{} 0 \end{pmatrix}\begin{pmatrix}x \\ \lambda \end{pmatrix}+ \begin{pmatrix}\underline{f'}(x_0) - \underline{A} x_0 \\ -E x_0 \end{pmatrix}\end{aligned}$$
  strongly approximates $\underline{{{\mathcal {F}}}}$ at $(x_0,q_0,\lambda _0)$, in the sense of Dontchev [10]. In the next two steps the strong regularity condition for $\underline{{{\mathcal {T}}}}$ will be verified.
(ii) (Existence). Let, at first, $(\beta _1, \beta _2) \in X^* \times W$ and consider $$\displaystyle {{\mathcal {T}}}\begin{pmatrix}x \\ \lambda \end{pmatrix}= \begin{pmatrix}\beta _1 \\ \beta _2 \end{pmatrix}$$ which is equivalent to,
  2.21 $$\begin{aligned} 0 \in \begin{pmatrix}a \\ -b \end{pmatrix}+ \begin{pmatrix}A &{} E^* \\ E &{} 0 \end{pmatrix}\begin{pmatrix}x \\ \lambda \end{pmatrix}+ \begin{pmatrix}\partial \mathbf {I}_C(x) \\ 0 \end{pmatrix}, \end{aligned}$$
  with $a = f'(x_0) - A x_0 - \beta _1, \ b = E x_0 + \beta _2$, and *A*, *E* defined in (2.4), (2.5). To solve (2.21) we consider,
  2.22 $$\begin{aligned} {\left\{ \begin{array}{ll} \min _{x \in C} \ \frac{1}{2}\langle Ax, x \rangle _{X^*,X} + \langle a, x \rangle _{X^*,X},\\ E x = b. \end{array}\right. } \end{aligned}$$
  This corresponds to (2.17) with ${\tilde{a}} = a$, ${\tilde{b}} = b$ and feasible set $\displaystyle S(\beta _2) = \{ x \in C: E x = b \}$. Clearly $\displaystyle x_0 \in S(0) = \{ x \in C: E x = E x_0 \}$. By (H2) and [16, Theorem I.2.8], there exists a neighborhood of the origin $\displaystyle {\tilde{V}} \subset X^* \times W$ such that $S(\beta _2)$ is not empty for all $(\beta _1, \beta _2) \in {\tilde{V}}$. Thus by Lemma 2.2 there exists a unique solution $x = x(\beta _1, \beta _2)$ to (2.22) for each $(\beta _1, \beta _2) \in {\tilde{V}}$. By [16, Theorem I.2.11, I.2.12, I.2.15], possibly after reducing ${\tilde{V}}$, these solutions depend Hölder continuously on $(\beta _1, \beta _2) \in {\tilde{V}} \subset X^* \times W$, with exponent $\frac{1}{2}$. The regular point condition for the solution $x(\beta _1, \beta _2)$ is
  $$\begin{aligned} 0 \in \text {int } E (C - x(\beta _1, \beta _2)) = \text {int } E (C - x_0) - \beta _2, \end{aligned}$$
  which is satisfied due to (H2), possibly after again shrinking ${\tilde{V}}$. Hence there exists a Lagrange multiplier $\lambda = \lambda (\beta _1, \beta _2)$ associated to $E x = b$, and (2.21) admits a unique solution $(x(\beta _1, \beta _2), \lambda (\beta _1, \beta _2))$ since it is the first order optimality condition for (2.17).
(iii) (Uniqueness and Lipschitz continuity) Let $(\beta _1, \beta _2) \in {\tilde{V}}$ and $(\hat{\beta _1}, \hat{\beta _2}) \in {\tilde{V}}$ with corresponding solutions $(x, \lambda ) \in X\times W^*$ and $({\hat{x}}, {\hat{\lambda }}) \in X\times W^*$. This implies that
  2.23 $$\begin{aligned} {\left\{ \begin{array}{ll} \langle a + Ax + E^*\lambda , c - x \rangle _{X^*,X} \ge 0, \ \forall c \in C,\\ Ex = b, \text { with } a = f'(x_0) - Ax_0 - \beta _1, \ b = Ex_0 + \beta _2, \end{array}\right. } \end{aligned}$$
  and
  2.24 $$\begin{aligned} {\left\{ \begin{array}{ll} \langle {\hat{a}} + A{\hat{x}} + E^*{\hat{\lambda }}, c - {\hat{x}} \rangle _{X^*,X} \ge 0,\ \forall c \in C,\\ E {\hat{x}} = {\hat{b}}, \text {with } {\hat{a}} = f'(x_0) - Ax_0 - \hat{\beta _1}, \ {\hat{b}} = Ex_0 + \hat{\beta _2}. \end{array}\right. } \end{aligned}$$
  By the first equations in (2.23) and (2.24) we obtain that
  2.25 $$\begin{aligned}&\langle a + A x + E^* \lambda , {\hat{x}} - x \rangle _{X^*,X} \ge 0, \langle {\hat{a}} + A {\hat{x}} + E^* {\hat{\lambda }} , x - {\hat{x}} \rangle _{X^*,X} \ge 0, \ x, {\hat{x}} \in C.\nonumber \\ \end{aligned}$$
  Combining these inequalities, we have that
  2.26 $$\begin{aligned} \langle a - {\hat{a}} + A (x - {\hat{x}}) + E^* (\lambda - {\hat{\lambda }}) , x - {\hat{x}} \rangle _{X^*,X} \le 0. \end{aligned}$$
  The second equalities in (2.23) and (2.24) imply that
  2.27 $$\begin{aligned} E(x - {\hat{x}}) = b - {\hat{b}}. \end{aligned}$$
  Let us set
  $$\begin{aligned} \delta x = {\hat{x}} - x, \ \delta \lambda = {\hat{\lambda }} - \lambda , \ \delta a = {\hat{a}} - a, \ \delta b = {\hat{b}} - b. \end{aligned}$$
  Then $\delta \beta _1 = - (\hat{\beta _1} - \beta _1)$ and $\delta \beta _2 = \hat{\beta _2} - \beta _2$, and (2.26), (2.27) result in
  2.28 $$\begin{aligned} \langle \delta x, A \delta x \rangle _{X,X^*} + \langle \delta \lambda , E \delta x \rangle _{W^*,W} - \langle \delta \beta _1, \delta x \rangle _{X^*,X} \le 0, \end{aligned}$$
  and
  2.29 $$\begin{aligned} E \delta x = \delta b. \end{aligned}$$
  By (H2) the operator *E* is surjective. Hence by the closed range theorem expressing $\delta x = \delta v + \delta w \in \text {ker } E + \text {range } E^*$ implies that $\displaystyle E \delta x = E \delta w = \delta \beta _2$. Again by the closed range theorem there exists $k_1 > 0$:
  2.30 $$\begin{aligned} \left\Vert \delta w\right\Vert _X \le k_1 \left\Vert \delta \beta _2\right\Vert _{W}. \end{aligned}$$
  From the first equation in (2.21) we have
  $$\begin{aligned} Ax + E^*\lambda -Ax_0 +f'(x_0) - \beta _1 \in - \partial {\mathbf {I}}_C(x). \end{aligned}$$
  Next we restrict the perturbation parameters to satisfy $(\beta _1,\beta _2) \in (\underline{X^*}\times W) \cap {{\tilde{V}}}$. Due to Assumptions (H4) and (H7) we have $(x, \lambda ) \in \underline{X} \times \underline{W^*}$,
  $$\begin{aligned} \underline{A} x + {\underline{E^*}} \lambda - \underline{A} x_0 + \underline{f'}(x_0) - \beta _1 \in \underline{X^*} \end{aligned}$$
  and hence
  $$\begin{aligned} \underline{A} x + {\underline{E^*}} \lambda - \underline{A} x_0 + \underline{f'}(x_0) - \beta _1 \in - \underline{\partial \mathbf {I}_C}(x). \end{aligned}$$
  The analogous equation holds with $(x,\lambda , \beta _1)$ replaced by $({{\hat{x}}}, {{\hat{\lambda }}}, {{\hat{\beta }}}_1)$. By (H3), (2.28) and Assumption (H7) we find
  2.31 $$\begin{aligned}&\kappa \left\Vert \delta v\right\Vert ^2 \nonumber \\&\quad \le \langle \delta v, A \delta v \rangle _{X, X^*} = \langle \delta x, A \delta x \rangle _{X, X^*} - 2 \langle \delta v, A \delta w \rangle _{X, X^*} - \langle \delta w, A \delta w \rangle _{X, X^*}\nonumber \\&\quad \le - \langle \delta \lambda , E \delta x \rangle _{W^*, W} + \langle \delta \beta _1, \delta x \rangle _{X, X^*} - 2 \langle \delta v, A \delta w \rangle _{X, X^*} - \langle \delta w, A \delta w \rangle _{X, X^*} \nonumber \\&\quad \le {{\tilde{k}}} \left\Vert \delta \lambda \right\Vert _{{\underline{W}}^*}\left\Vert \delta \beta _2\right\Vert _W + \left\Vert \delta \beta _1\right\Vert _{X^*} \left\Vert \delta x\right\Vert _{X} + \left\Vert A\right\Vert \left\Vert \delta w\right\Vert _{X} \left( 2 \left\Vert \delta v\right\Vert _X + \left\Vert \delta w\right\Vert _X \right) \nonumber \\&\quad \le {{\tilde{k}}} k ( \left\Vert \delta w\right\Vert _X + \left\Vert \delta v\right\Vert _X + \left\Vert \delta \beta \right\Vert _{{\underline{X}}^*\times W} ) \left\Vert \delta \beta _2\right\Vert _W\nonumber \\&\quad \quad + ( \left\Vert \delta \beta _1\right\Vert _{ X^*} + 2 \left\Vert A\right\Vert \left\Vert \delta w\right\Vert _X) (\left\Vert \delta v\right\Vert _X + \left\Vert \delta w\right\Vert _X), \end{aligned}$$
  where ${{\tilde{k}}}$ denotes the embedding constant of ${\underline{W}}^*$ into $W^*$. Using (2.30) and rearranging terms there exists a constant $k_2 > 0$ such that
  2.32 $$\begin{aligned} \left\Vert \delta v\right\Vert _X \le k_2 \left( \left\Vert \delta \beta _1\right\Vert _{\underline{X}^*} + \left\Vert \delta \beta _2\right\Vert _W \right) . \end{aligned}$$
  Applying (2.30) again this implies the existence of $$ k_3$$ such that
  2.33 $$\begin{aligned} \left\Vert \delta x\right\Vert _X \le k_3 \left( \left\Vert \delta \beta _1\right\Vert _{\underline{X}^*} + \left\Vert \delta \beta _2\right\Vert _W \right) \text { for all } (\beta _1, \beta _2) \in (\underline{X^*} \times W)\cap {{\tilde{V}}}. \end{aligned}$$
  Another application of (H7) and (2.33) imply the existence of a constant $k_4$ and a neighborhood ${{\hat{V}}}$ of the origin in $\underline{X^*} \times W$ such that the desired Lipschitz stability estimate for $(\underline{{{\mathcal {T}}}})^{-1}$
  2.34 $$\begin{aligned} \left\Vert \delta x\right\Vert _{\underline{X}} + \left\Vert \delta \lambda \right\Vert _{\underline{W^*}} \le k_4 \left( \left\Vert \delta \beta _1\right\Vert _{\underline{X^*}} + \left\Vert \delta \beta _2\right\Vert _W \right) \text { for all } (\beta _1, \beta _2) \in {{\hat{V}}} \subset \underline{X^*} \times W \end{aligned}$$
  holds.
(iv) As a consequence of the previous two steps $\underline{{{\mathcal {T}}}}$ is strongly regular at $(x_0, q_0, \lambda _0)$. Together with step (i), Dontchev’s theorem is applicable [10, Theorem 2.4, Remark 2.5], and (2.15), and (2.16) follow.
(v) (Local solution to ($P_q$)) Now we show that there exists a neighborhood ${\tilde{N}}$ of $q_0$ such that for $q \in {\tilde{N}}$ the second order sufficient optimality condition is satisfied at *x*(*q*), so that *x*(*q*) is a local solution of ($P_q$) by e.g. [16, Theorem 2.12, p. 42]. Due to (H3) and regularity of *f*, *e* we obtain
  2.35 $$\begin{aligned} {{\mathcal {L}}}''(x(q),q,\lambda (q))(h,h) \ge \frac{\kappa }{2} \left\Vert h\right\Vert ^2, \text {for all } h \in \text {ker } E, \ \text {if } q \in N(q_0).\quad \, \end{aligned}$$
  Let us define $\displaystyle E_q = (e_y(x(q),q))$ for $q \in N(q_0)$. By the surjectivity of $E_{q_0}$ and regularity of *e* there exists a neighborhood ${\tilde{N}} \subset N(q_0)$ such that $E_q$ is surjective for all $q \in {\tilde{N}}$. Here we also use continuity of $q \mapsto e_y(x(q), q)$ from $P \rightarrow W$ at $q_0$, which follows from (H1) and the continuity of $q \rightarrow x(q)$ at $q_0$. Consequently exist $\delta _0, \gamma > 0$ such that
  2.36 $$\begin{aligned} {{\mathcal {L}}}''(x(q), q, \lambda (q))(h + z,h + z) \ge \delta _0 \left\Vert h + z\right\Vert ^2,\ \text {for all } h \in \text {ker } E, z \in X \end{aligned}$$
  satisfying $\left\Vert z\right\Vert \le \gamma \left\Vert h\right\Vert$ by [16, Lemma 2.13, p. 43]. Let us define the orthogonal projection onto ker$\ E_q$ given by $\displaystyle P_{\text {ker } E_q} = I - E_q^*(E_qE_q^*)^{-1}E_q$. We choose ${\tilde{N}}$ so that
  $$\begin{aligned} \left\Vert E_q^*(E_qE_q^*)^{-1}E_q - E_{q_0}^*(E_{q_0} E_{q_0}^*)^{-1}E_{q_0}\right\Vert \le \frac{\gamma }{1 + \gamma } \end{aligned}$$
  for all $q \in {\tilde{N}}$. For $x \in \text {ker } E_q$, we have $x = h + z$ for $h \in \text {ker } E, \ z \in (\text {ker } E)^{\perp }$ and $\left\Vert x\right\Vert ^2 = \left\Vert h\right\Vert ^2 + \left\Vert z\right\Vert ^2$. Thus,
  $$\begin{aligned} \left\Vert z\right\Vert \le \left\Vert E_q^*(E_qE_q^*)^{-1}E_q x - E_{q_0}^*(E_{q_0} E_{q_0}^*)^{-1}E_{q_0} x\right\Vert \le \frac{\gamma }{1 + \gamma } \big ( \left\Vert h\right\Vert + \left\Vert z\right\Vert \big ) \end{aligned}$$
  and hence $\left\Vert z\right\Vert \le \gamma \left\Vert h\right\Vert$. From (2.35) this implies
  $$\begin{aligned} {{\mathcal {L}}}''(x(q), q, \lambda (q)) \ge \delta _0 \left\Vert x\right\Vert ^2, \ \text {for all } x \in \text {ker } E. \end{aligned}$$
  This concludes the proof. $\square$

## Differentiability of Value Function for Optimal Stabilization Subject to Semi-linear Parabolic Equations

Here we describe the optimal control problems which we shall analyze and state the main results.

### Notation

Let $\Omega$ be an open connected bounded subset of ${\mathbb {R}}^d$ with a Lipschitz continuous boundary $\Gamma$. The associated space-time cylinder is denoted by $Q = \Omega \times (0,\infty )$ and the associated lateral boundary by $\Sigma = \Gamma \times (0,\infty )$. We define the Hilbert spaces

$$\begin{aligned} Y = L^2(\Omega ), \quad V = H^1_0(\Omega ), \text { and }\quad U = L^2(0,\infty ;\,{{\mathcal {U}}}), \end{aligned}$$

where ${{\mathcal {U}}}$ is a Hilbert space which will be identified with its dual. Observe that the embedding $V \subset Y$ is dense and compact. Further $V \subset Y \subset V^*$, is a Gelfand triple. Here $V^*$ denotes the topological dual of *V* with respect to the pivot space *Y*. For any $T \in (0, \infty )$ we define the space

$$\begin{aligned} W(0,T) = \bigg \{ y \in L^2(0,T;V); \ \frac{dy}{dt} \in L^2(0,T;V^*) \bigg \}, \end{aligned}$$

endowed with the norm

$$\begin{aligned} \left\Vert y\right\Vert _{W(0,T)} = \left( \left\Vert y\right\Vert _{L^2(0,T;V)}^2 + \left\Vert \frac{dy}{dt}\right\Vert _{L^2(0,T;V^*)}^2 \right) ^{{}^{1}\!/_{2}}. \end{aligned}$$

For $T = \infty$, we write $W_{\infty }$ and $I = (0,\infty )$. We further set $W^0_{\infty }=\{y\in W_{\infty }: y(0)=0 \}$. We also set

$$\begin{aligned} W(T,\infty ) = \bigg \{ y \in L^2(T,\infty ;V); \ \frac{dy}{dt} \in L^2(T,\infty ;V^*) \bigg \}. \end{aligned}$$

We shall frequently use that $W_\infty$ embeds continuously into $C([0,\infty ),Y)$, see e.g. [17, Theorem 4.2] and that $\displaystyle \lim _{t \rightarrow \infty } y(t) = 0$, for $y \in W_\infty$, see e.g. [9]. The set of admissible controls $U_{ad}$ is chosen to be

3.1 $$\begin{aligned} U_{ad} \subset \{u \in U: \Vert u(t)\Vert _{{{\mathcal {U}}}}\le \eta , \text { for } a.e. \ t>0 \} \end{aligned}$$

where $\eta$ is a positive constant. We further set ${{\mathcal {U}}}_{ad} = \{v \in {{\mathcal {U}}}: \Vert v \Vert _{{{\mathcal {U}}}}\le \eta \}$ and denote by $\displaystyle {\mathbb {P}}_{{{\mathcal {U}}}_{ad}}$ the projection of ${{\mathcal {U}}}$ on ${{\mathcal {U}}}_{ad}$. For this choice of admissible controls the dynamical system can be stabilized for all sufficiently small initial conditions in *Y*, see Corollary 4.3 and Remark 4.1.

For $\delta > 0$ and ${\bar{y}} \in Y$, we define the open neighborhoods $B_Y(\delta ) = \left\{ y \in Y: \left\Vert y\right\Vert _Y < \delta \right\} ,$ and $\quad B_Y({\bar{y}}, \delta ) = \left\{ y \in Y: \left\Vert y - {\bar{y}}\right\Vert _Y < \delta \right\}$.

### Problem Formulation and Assumptions

We focus on the stabilization problem for an abstract semi-linear parabolic equation formulated as infinite horizon optimal control problem under control constraints:

3.2a $$\begin{aligned} ({\mathcal {P}}) \qquad {\mathcal {V}}(y_0) = \min _{ (y, u)\in W_\infty \times U_{ad}} \ J(y,u)&= \min _{ (y, u)\in W_\infty \times U_{ad}} \ \frac{1}{2} \int _{0}^{\infty } \left\Vert y(t)\right\Vert ^2_Y dt\nonumber \\&\quad + \frac{\alpha }{2} \int _{0}^{\infty } \left\Vert u(t)\right\Vert ^2_{{{\mathcal {U}}}} dt, \end{aligned}$$

subject to the semilinear parabolic equation

3.2b $$\begin{aligned} y_t&= {{\mathcal {A}}}y + {{\mathcal {F}}}(y) + B u \quad \text { in } L^2(I; V^*) \end{aligned}$$

3.2c $$\begin{aligned} y(x,0)&= y_0 \quad \text { in } Y. \end{aligned}$$

Throughout ${{\mathcal {F}}}$ is the substitution operator associated to a mapping ${\mathfrak {f}}:{\mathbb {R}}\rightarrow {\mathbb {R}}$ so that $({{\mathcal {F}}}y)(t)={\mathfrak {f}} (y(t))$. Sufficient conditions which guarantee the existence of solutions to (3.2b), (3.2c), as well as solutions $(\bar{y}, \bar{u})$ to (${\mathcal {P}}$), for $y_0\in Y$ sufficiently small, will be given below. We shall also make use of the adjoint equation associated to an optimal state ${\bar{y}}$, given by

3.2d $$\begin{aligned} -p_t - {{\mathcal {A}}}^* p - {{\mathcal {F}}}'(\bar{y})^* p = -\bar{y} \quad \text { in } L^2(I;V^*). \end{aligned}$$

Its adjoint state *p* which will be considered in $L^2(I;V)$ or in $W_\infty$. The following assumption will be essential.

#### Assumptions A

**A1**: The operator ${{\mathcal {A}}}$ with domain ${{\mathcal {D}}}({{\mathcal {A}}})\subset Y$ and range in *Y*, generates a strongly continuous analytic semigroup $\displaystyle e^{{{\mathcal {A}}}t}$ on *Y* and can be extended to ${{\mathcal {A}}}\in {{\mathcal {L}}}(V, V^*)$.
**A2**: $B \in {{\mathcal {L}}}({{\mathcal {U}}},Y)$ and there exists a stabilizing feedback operator $K \in {{\mathcal {L}}}(Y,{{\mathcal {U}}})$ such that the semigroup $\displaystyle e^{({{\mathcal {A}}}-BK)t}$ is exponentially stable on *Y*.
**A3**: The nonlinearity $\displaystyle {{\mathcal {F}}}: W_\infty \rightarrow L^2(I; V^*)$ is twice continuously Fréchet differentiable, with second Fréchet derivative ${{\mathcal {F}}}''$ bounded on bounded subsets of $W_\infty$, and ${{\mathcal {F}}}(0) = 0$.
**A4**: ${{\mathcal {F}}}:W(0,T) \rightarrow L^1(0,T;{{\mathcal {H}}}^*)$ is weak-to-weak continuous for every $T>0$, for some Hilbert space ${{\mathcal {H}}}$ which embeds densely in *V*.
  Note that $\displaystyle \left( L^1(0,T; {{\mathcal {H}}}^*) \right) ^* = L^{\infty }(0,T; {{\mathcal {H}}})$, see [11, Theorem 7.1.23(iv), p. 164]. Moreover, $L^{\infty }(0,T; {{\mathcal {H}}})$ is dense in $L^2(0,T; V)$, see [21, Lemma A.1, p. 2231].
**A5**: $${{\mathcal {F}}}'(\bar{y})\in {{\mathcal {L}}}{(L^2(I;V), L^2(I;V^*))}$$.

##### Remark 3.1

The requirement that ${\mathcal {F}}(0) = 0$ in (A2) is consistent with the fact that we focus on the stabilization problem with 0 as steady state for (3.2b). Without loss of generality we further assume that

3.3 $$\begin{aligned} {\mathcal {F}}'(0) = 0, \end{aligned}$$

which can always be achieved by making ${\mathcal {F}}'(0)$ to be perturbation of ${\mathcal {A}}$.

##### Remark 3.2

Let us assume that (A3) holds. Then in view of the fact that ${{\mathcal {F}}}$ is a substitution operator we have $[{{\mathcal {F}}}'(y) v](t)= {\mathfrak {f}}'(y(t))v(t)$ for *y* and *v* in $W_\infty$, and ${{\mathcal {F}}}'(y)\in {{\mathcal {L}}}(W_\infty ,L^2(I;V^*))$. Its adjoint $[{{\mathcal {F}}}'(y)^* v](t)= {\mathfrak {f}}'(y(t))v(t)$, for $v\in L^2(I;V)$, satisfying ${{\mathcal {F}}}'(y)^*\in {{\mathcal {L}}}(L^2(I;V),W_\infty ^*)$. It has a natural restriction to an operator $\underline{{{\mathcal {F}}}'(y)^*} \in {{\mathcal {L}}}(W_\infty ,L^2(I;V^*))$. With (A3) holding it is differentiable and $[\underline{{{\mathcal {F}}}'(y)}^*]'$ is a bilinear form on $W_\infty \times W_\infty$ with values in $L^2(I;V^*)$. - For examples of functions ${{\mathcal {F}}}$ which satisfy $\mathbf{A4}$ we refer to see Sect. 6.

#### Abstract Setup

Here we relate problem (${\mathcal {P}}$) to the abstract problem ($P_q$), which is used with the following spaces:

3.4 $$\begin{aligned} X= & {} W_{\infty } \times U, \; W = L^2(I;V^*) \times Y, \; P = Y, \;\nonumber \\ C= & {} U_{ad}, \; X^* = W_{\infty }^* \times U, \; W^* = L^2(I;V) \times Y,\nonumber \\ \underline{X}= & {} W_{\infty } \times (U \cap C({\bar{I}};{{\mathcal {U}}})), \quad \underline{X^*} = L^2(I;V^*) \times (U \cap C({\bar{I}};{{\mathcal {U}}})), \quad \underline{W^*} = \widetilde{W}_{\infty },\nonumber \\ \end{aligned}$$

where $I=(0,\infty )$, and ${\widetilde{{{\mathcal {W}}}}} = \{(\varphi ,\varphi (0)):\varphi \in W_\infty \}$, endowed with the norm of $W_{\infty }$. At times we identify ${\widetilde{{{\mathcal {W}}}}}$ with $W_{\infty }$. We recall that the dual space of $\displaystyle W_\infty = L^2(I;V) \cap W^{1,2}(I;V^*)$ is $\displaystyle W^*_\infty = L^2(I;V^*) + (W^{1,2}(I;V^*))^*$, endowed with the norm $\displaystyle \left\Vert z\right\Vert _{ W^*_\infty } = \inf _{ z= z_1 + z_2} \left\Vert z_1\right\Vert _{L^2(I;V^*)} + \left\Vert z_2\right\Vert _{W^{1,2}(I;V^*)^*}$, where $\displaystyle z_1 \in L^2(I;V^*), z_2 \in (W^{1,2}(I;V^*))^*$.

To express ($P_q$) for the present case, we set $x = (y,u) \in W_{\infty } \times U$, and the parameter *q* becomes the initial condition $y_0 \in Y$. Further $f: W_{\infty } \times U \longrightarrow {\mathbb {R}}$ is given by

3.5 $$\begin{aligned} f(y,u) = \frac{1}{2}\int _{0}^{\infty } \left\Vert y(t)\right\Vert ^2_{Y} dt + \frac{\alpha }{2}\int _{0}^{\infty } \left\Vert u(t)\right\Vert ^2_{{{\mathcal {U}}}} dt, \end{aligned}$$

and $$e(x,q) = e(y,u,y_0)$$ is

3.6 $$\begin{aligned} e(y,u,y_0) = \begin{pmatrix}y_t - {{\mathcal {A}}}y - {{\mathcal {F}}}(y) - Bu \\ y(0) - y_0 \end{pmatrix}: W_{\infty } \times U \times Y \longrightarrow L^2(I; V^*) \times Y \end{aligned}$$

By (A3) the mapping *e* is Fréchet differentiable with respect to $\displaystyle x = (y,u) \in W_{\infty } \times U$ and thus for $(y,u,y_0) \in W_{\infty } \times U \times Y$ we have

3.7 $$\begin{aligned} e'(y,u,y_0)(v,w) = \begin{pmatrix}v_t - {{\mathcal {A}}}v - {{\mathcal {F}}}'(y) v - B w\\ v(0, \cdot ) \end{pmatrix}: W_{\infty } \times U \longrightarrow L^2(I; V^*) \times Y. \end{aligned}$$

The Lagrange functional ${{\mathcal {L}}}: W_\infty \times U \times Y \times L^2(I;V) \times Y \longrightarrow {\mathbb {R}}$ corresponding to our optimal control problem is given by

$$\begin{aligned}&{{\mathcal {L}}}(y, u, y_0, p,p_1) = J(y,u) + \int _0^{\infty } \langle p, y_t - {{\mathcal {A}}}y - {{\mathcal {F}}}(y) - Bu \rangle _{V, V^*}dt + (p_1,y(0) - y_0)_Y, \end{aligned}$$

where $(p,p_1) \in L^2(I;V) \times Y$ corresponds to the abstract Lagrange multiplier $\lambda \in W^*$.

In the remainder of this subsection we specify the mappings ${{\mathcal {T}}}$ and $\underline{{{\mathcal {T}}}}$ for problem (${\mathcal {P}}$). This will facilitate the proofs of the main results further below.

At first we take a closer look to the adjoint $\displaystyle \widetilde{E}^* := e'(y,u,y_0)^* \in {{\mathcal {L}}}(L^2(I;V) \times Y,W^*_\infty \times U)$ at a generic element $(y,u,y_0) \in W_{\infty } \times U \times Y$. It is characterized by the property that for all $(v,w) \in W_\infty \times U,\ (p,p_1) \in L^2(I;V) \times Y$ we have

$$\begin{aligned} \langle \widetilde{E}(v,w), (p,p_1) \rangle _{L^2(I;V^*) \times Y, L^2(I;V) \times Y} = \langle v, \widetilde{E}_1^*(p,p_1) \rangle _{W_\infty , W^*_\infty } + ( w, \widetilde{E}_2^*(p,p_1) )_{U} \end{aligned}$$

where

$$\begin{aligned} \langle v, \widetilde{E}_1^*(p,p_1) \rangle _{W_\infty , W^*_\infty } = \langle v_t - {{\mathcal {A}}}v -{{\mathcal {F}}}'(y)v, p \rangle _{L^2(I;V^*),L^2(I;V)} + ( v(0), p_1 )_Y, \end{aligned}$$

and

$$\begin{aligned} ( w, \widetilde{E}_2^*(p,p_0) )_{U} = - ( w, B^*p )_U. \end{aligned}$$

If for some ${\tilde{\beta }}_1 \in L^2(I;V^*)$ the pair $(p,p_1) \in L^2(I;V) \times Y$ is a solution to ${{\tilde{E}}}_1^*(p,p_1)= \tilde{\beta }_1$ then for all $v \in W_{\infty }$:

3.8 $$\begin{aligned}&\langle v_t - {{\mathcal {A}}}v -{{\mathcal {F}}}'(y)v, p \rangle _{L^2(V^*),L^2(V)} + ( v(0), p_1 )_Y - ( w, B^*p )_U\nonumber \\&\quad = \langle \tilde{\beta }_1,v\rangle _{L^2(I;V^*),L^2(I;V)}. \end{aligned}$$

Now we assume that ${{\mathcal {F}}}'(y)$ is not only an element of $\displaystyle {{\mathcal {L}}}(W_{\infty },L^2(I;V^*))$ but rather that it can be extended to an operator ${{{\mathcal {F}}}'(y)} \in {{\mathcal {L}}}(L^2(I;V),L^2(I;V^*))$. This is guaranteed by (A5) at minimizers ${\bar{y}}$. Then (3.8) implies that $p \in W_{\infty }$, and hence $p_1 \in C(I;Y)$ and $p_1 = p(0)$, see Proposition 1. In particular $(p,p_1) = (p,p(0)) \in \widetilde{W}_{\infty }$, and (3.8) can equivalently be expressed as

3.9 $$\begin{aligned} \langle v, \widetilde{E}_1^*(p,p_1) \rangle _{L^2(I;V),L^2(I;V^*)}= & {} \langle v_t - {{\mathcal {A}}}v -{{\mathcal {F}}}'(y)v, p \rangle _{L^2(I;V^*),L^2(I;V)}\nonumber \\= & {} \langle v, \widetilde{\beta }_1 \rangle _{L^2(I;V),L^2(I;V^*)}, \end{aligned}$$

for all $v \in L^2(I;V)$, where we assumed that $\widetilde{\beta }_1 \in L^2(I;V^*)$. Conversely, of course, if $p \in \widetilde{W}_{\infty }$, then $\displaystyle \widetilde{E}^*_1 (p,p(0)) = -p_t - {{\mathcal {A}}}^*p - {{\mathcal {F}}}'(y)^*p \in L^2(I;V^*)$.

From now on, let $q_0 = \bar{y}_0$ denote a reference (or nominal) parameter with associated solution $x_0 = (\bar{y}, \bar{u})$. In Proposition 1 we shall argue that the regular point condition Assumption (H2) is satisfied and that consequently there exists a Lagrange multiplier $(\bar{p}, \bar{p}_1)$ such that the pair $(x_0, \lambda _0) = (\bar{y}, \bar{u}, \bar{p},\bar{p}_1)$ satisfies (2.3). Moreover, it will turn out that ${\bar{p}} \in W_\infty$, ${\bar{p}}_1 = p(0)$, and that ${\bar{u}} \in U \cap C(I;{{\mathcal {U}}})$. For convenience let us present (2.3) for the present case

3.10 $$\begin{aligned} 0 \in {\left\{ \begin{array}{ll} \bar{y} + E^*_1({\bar{p}}, {\bar{p}}(0)),\\ \alpha {\bar{u}} - B^* {\bar{p}} + \partial \mathbf {I}_{U_{ad}}({\bar{u}}),\\ {\bar{y}}_t - {{\mathcal {A}}}{\bar{y}} - {{\mathcal {F}}}({\bar{y}}) - B {\bar{u}},\\ {{\bar{y}}(0)} - y_0, \end{array}\right. } \end{aligned}$$

where $$\displaystyle E = \begin{pmatrix}E_1 \\ E_2 \end{pmatrix}= e'({\bar{y}}, {\bar{u}}, {\bar{y}}_0)$$. We stress that while the Lagrange multiplier ${\bar{p}}$ belongs to $W_{\infty }$, the operator $E^*_1$ in (3.9) is still considered as an element of ${{\mathcal {L}}}(L^2(I;V) \times Y, W^*_{\infty })$.

We are now prepared to specify the multivalued operators

3.11 $$\begin{aligned} {{\mathcal {T}}}:&W_{\infty } \times U \times L^2(I;V) \times Y \longrightarrow W_{\infty }^* \times U \times L^2(I;V^*) \times Y, \quad \text {and} \end{aligned}$$

3.12 $$\begin{aligned} \underline{{{\mathcal {T}}}}:&W_{\infty } \times (U \cap C(I;{{\mathcal {U}}})) \times \widetilde{W}_{\infty } \longrightarrow L^2(I;V^*) \times (U \cap C(I;{{\mathcal {U}}})) \times L^2(I;V^*) \times Y \end{aligned}$$

corresponding to (2.7) and (2.14) by

3.13 $$\begin{aligned} {{\mathcal {T}}}\begin{pmatrix}y \\ u \\ p \\ p_1 \end{pmatrix}= \begin{pmatrix}E^*_1(p,p_1) + y - [{{\mathcal {F}}}'(\bar{y})^*p]'(y - \bar{y})\\ \alpha u - B^* p\\ y_t - {{\mathcal {A}}}y - Bu - {{\mathcal {F}}}'(\bar{y})(y - \bar{y}) - {{\mathcal {F}}}(\bar{y})\\ y(0) - {\bar{y}_0} \end{pmatrix}+ \begin{pmatrix}0 \\ \partial \mathbf {I}_{U_{ad}}(u) \\ 0 \\ 0 \end{pmatrix}, \end{aligned}$$

and

3.14 $$\begin{aligned} \underline{{{\mathcal {T}}}} \begin{pmatrix}y \\ \underline{u} \\ \underline{p} \\ \underline{p}(0) \end{pmatrix}= & {} \begin{pmatrix}- \underline{p}_t - {{\mathcal {A}}}^* \underline{p} - \underline{{{\mathcal {F}}}'(\bar{y})^*}\, \underline{p} + y - [\underline{{{\mathcal {F}}}'(\bar{y})^*}\,\underline{\bar{p}}]'(y - \bar{y})\\ \alpha \underline{u} - B^* \underline{p}\\ y_t - {{\mathcal {A}}}y - B\underline{u} - {{\mathcal {F}}}'(\bar{y})(y - \bar{y}) - {{\mathcal {F}}}(\bar{y})\\ y(0) - {\bar{y}_0} \end{pmatrix}\nonumber \\&+ \begin{pmatrix}0 \\ \underline{\partial \mathbf {I}_{U_{ad}}}(\underline{u}) \\ 0 \\ 0 \end{pmatrix}. \end{aligned}$$

where

3.15 $$\begin{aligned} \underline{\partial \mathbf {I}_{U_{ad}}}(\underline{u}) = \left\{ \widetilde{u} \in U \cap C(I;{{\mathcal {U}}}): ( \widetilde{u}(t), v - \underline{u}(t) )_{{\mathcal {U}}}\le 0, \forall t \in I, v \in B_{\eta }(0) \right\} , \end{aligned}$$

with $\displaystyle B_{\eta }(0) = \left\{ v \in {{\mathcal {U}}}; \left\Vert v\right\Vert _{{{\mathcal {U}}}} \le \eta \right\}$. In (3.14), we underline the elements which are taken from different domains when compared to (3.13). The range of the first two coordinates of $\underline{{{\mathcal {T}}}}$ is smaller than that of ${{\mathcal {T}}}$. Accordingly we can make use of (3.9) when moving from the first row of (3.13) to the first row of (3.14).

For convenience for the subsequent work we recall that the strong regularity condition introduced below (2.14) requires us to find neighborhoods of 0 and $(\bar{y}, \bar{u}, \bar{p},\bar{p}(0))$ of the form ${\hat{V}} \subset L^2(I;V^*) \times (U \cap C(\overline{I};{{\mathcal {U}}})) \times L^2(I;V^*) \times Y$ and ${\hat{U}} \subset W_{\infty } \times (U \cap C(\overline{I};{{\mathcal {U}}})) \times {\widetilde{W}}_{\infty }$, such that for all $\varvec{\beta } = (\beta _1, \beta _2, \beta _3, \beta _4) \in {{\hat{V}}}$ the equation

3.16 $$\begin{aligned} \underline{{{\mathcal {T}}}} \left( y, u, \underline{p}, \underline{p}(0) \right) ^T = \left( \beta _1, \beta _2, \beta _3, \beta _4 \right) ^T \end{aligned}$$

admits a unique solution $(y,\underline{u},\underline{p},\underline{p}(0)) \in {{\hat{U}}}$ depending Lipschitz-continuously on $\varvec{\beta }$.

##### Remark 3.3

We observe that as a consequence of (A3) and Remark 3.2 the operator $\underline{{{\mathcal {T}}}}$ is continuous.

Subsequently we shall frequently refrain from the underline-notation since the meaning should be clear from the context.

### Main Theorems

In this subsection, we present the main theorems of this paper. The first theorem asserts local continuous differentiability of the value function ${\mathcal {V}}$ w.r.t. $y_0$, with $y_0$ small enough. The second theorem establishes that ${\mathcal {V}}$ satisfies the HJB equation in the classical sense. The proof of the first theorem is based on Theorem 2.1. It will be given in Sect. 4. For this purpose it will be shown that assumptions A imply (H1)–(H7). Moreover we need to assert the underlying assumption that problem (${\mathcal {P}}$) is well-posed. This will lead to a smallness assumption on the initial states $y_0$. Consequently it would suffice to assume that (A3) and (A4) only hold locally in the neighborhood of the origin. Concerning (A5) observe that it is not implied by (A3). It is vacuously satisfied for ${\bar{y}}=0$, which is the case for $y_0=0$, since then ${\mathcal {F}}'(0) = 0$, see (3.3).

We invoke Theorem 2.1 to assert the Lipschitz continuity of the state, the adjoint state, and the control with respect to the initial condition $y_0 \in Y$ in the neighborhood of a locally optimal solution $({\bar{y}}, {\bar{u}})$ corresponding to a sufficiently small reference initial state $\bar{y}_0$. This will imply the differentiability of the value function associated to local minima. We shall refer to the value function associated to local minima as ’local value function’.

#### Theorem 3.1

Let the assumptions (A) hold. Then associated to each local solution $({\bar{y}}(y_0), {\bar{u}}(y_0))$ of (${\mathcal {P}}$) there exists a neighborhood of $U(y_0)$ such that the local value function ${{\mathcal {V}}}: U(y_0)\subset Y \rightarrow {\mathbb {R}}$ is continuously differentiable, provided that $y_0$ is sufficiently close to the origin in *Y*.

To obtain a HJB equation we require additionally that $t \rightarrow ({{\mathcal {F}}}({\bar{y}}))(t)$ is continuous with values in *Y* for global solutions $({\bar{y}}, {\bar{u}})$ to (${\mathcal {P}}$), with $y_0\in {{\mathcal {D}}}({{\mathcal {A}}})$. In view of the fact that for $y_0\in V$ we can typically expect that the solutions of semilinear parabolic equations satisfy $y \in L^2(I;{{\mathcal {D}}}({{\mathcal {A}}})) \cap W^{1,2}(I;Y) \subset C([0,\infty ),V)$ this is not a restrictive assumption beyond that what is already assumed in (A3).

#### Theorem 3.2

Let the assumptions (A) hold, and let $(\bar{y}(y_0), {\bar{u}}(y_0))$ denote a global solution of (${\mathcal {P}}$), for $y_0\in {{\mathcal {D}}}({{\mathcal {A}}})$ with sufficiently small norm in *Y*. Assume that there exists $T_{y_0}>0$ such that ${{\mathcal {F}}}({\bar{y}})\in C([0,T_{y_0});Y)$. Then the following Hamilton–Jacobi–Bellman equation holds at $y_0$:

3.17 $$\begin{aligned} {\mathcal {V}}'(y)({{\mathcal {A}}}y + {{\mathcal {F}}}(y))+ & {} \frac{1}{2} \left\Vert y\right\Vert ^2_Y + \frac{\alpha }{2} \left\Vert {\mathbb {P}}_{{\mathcal {U}}_{ad}} \left( -\frac{1}{\alpha }B^*{\mathcal {V}}'(y) \right) \right\Vert ^2_Y\nonumber \\&+ \left\langle B^* {\mathcal {V}}'(y),{\mathbb {P}}_{{\mathcal {U}}_{ad}} \left( -\frac{1}{\alpha }B^*{\mathcal {V}}'(y) \right) \right\rangle _Y = 0. \end{aligned}$$

Moreover the optimal feedback law is given by

3.18 $$\begin{aligned} {{\bar{u}}}(0) = {\mathbb {P}}_{{\mathcal {U}}_{ad}} \left( -\frac{1}{\alpha } B^*{\mathcal {V}}'({{\bar{y}}}(0)) \right) . \end{aligned}$$

The condition on the smallness of $y_0$ will be discussed in Remark 4.2. Roughly it involves well-posedness of the optimality system and second order sufficient optimality at local solutions. A more detailed, respectively stronger statement of Theorem 3.1 and Theorem 3.2, will be given in Theorems 4.10 and 5.1. The regularity assumptions ${{\mathcal {F}}}({\bar{y}})\in C([0,T_{y_0});Y)$ of Theorem 3.2 will be addressed in Sect. 6.

## Proof of Theorem 3.1

In this section we give the proof for Theorem 3.1. Many of the technical difficulties arise from the fact that we are working with an infinite horizon optimal control problem. In this respect we can profit from techniques which were developed in [6], which, however, do not include the case of constraints on the norm. Throughout we assume that assumptions (A1) - (A4) hold.

### Well-posedness of Problem (${\mathcal {P}}$)

Here we prove well-posedness for (${\mathcal {P}}$) with small initial data. First, we recall two consequences of the assumption that ${{\mathcal {A}}}$ is the generator of an analytic semigroup.

#### Consequence 1

Since ${{\mathcal {A}}}$ generates a strongly continuous analytic semigroup on *Y*, there exist $\rho \ge 0$ and $\theta > 0$ such that

$$\begin{aligned} \langle (\rho I - {{\mathcal {A}}})v, v \rangle _{V^*,V} \ge \theta \left\Vert v\right\Vert ^2_V \end{aligned}$$

See [1, Part II, Chaptor 1, p. 115], [22, Theorem 4.2, p. 14].

#### Consequence 2

For all $y_0 \in Y, f \in L^2(0,T; V^*)$, and $T > 0$, there exists a unique solution $y \in W(0,T)$ to

4.1 $$\begin{aligned} {\dot{y}} = {{\mathcal {A}}}y + f, \quad y(0) = y_0. \end{aligned}$$

Furthermore, *y* satisfies

4.2 $$\begin{aligned} \left\Vert y\right\Vert _{W(0,T)} \le c(T) \Big ( \left\Vert y_0\right\Vert _{Y} + \left\Vert f\right\Vert _{L^2(0,T; V^*)} \Big ) \end{aligned}$$

for a continuous function *c*. Assuming that $y \in L^2(0, \infty ; Y)$, consider the equation

$$\begin{aligned} {\dot{y}} = \underbrace{({{\mathcal {A}}}- \rho I)y}_{{{\mathcal {A}}}_{\rho }} + \underbrace{\rho y + f}_{f_{\rho }}, \quad y(0) = y_0, \end{aligned}$$

where $f_{\rho } \in L^2(I; V^*)$. Then the operator ${{\mathcal {A}}}_{\rho }$ generates a strongly continuous analytic semigroup on *Y* which is exponentially stable, see [1, p 115, Theorem II.1.2.12]. It follows that $y \in W_{\infty }$, that there exists $M_{\rho }$ such that

4.3 $$\begin{aligned} \left\Vert y\right\Vert _{W_{\infty }} \le M_{\rho } \Big ( \left\Vert y_0\right\Vert _{Y} + \left\Vert f_{\rho }\right\Vert _{L^2(I; V^*)} \Big ), \end{aligned}$$

and that *y* is the unique solution to (4.1) in $W_{\infty }$, see [6, Sect. 2.2] .

#### Lemma 4.1

There exists a constant $C > 0$, such that for all $\delta < (0,1]$ and for all $y_1$ and $y_2$ in $W_{\infty }$ with $\displaystyle \left\Vert y_1\right\Vert _{W_{\infty }} \le \delta$ and $\displaystyle \left\Vert y_2\right\Vert _{W_{\infty }} \le \delta$, it holds that

4.4 $$\begin{aligned} \left\Vert {{\mathcal {F}}}(y_1) - {{\mathcal {F}}}(y_2)\right\Vert _{L^2(I; V^*)} \le \delta C \left\Vert y_1 - y_2\right\Vert _{W_{\infty }}. \end{aligned}$$

#### Proof

Let $y_1, y_2$ be as in the statement of the lemma. Using **A3** and Remark 3.1 we obtain the estimate

$$\begin{aligned}&\left\Vert {{\mathcal {F}}}(y_1) - {{\mathcal {F}}}(y_2)\right\Vert _{L^2(I,V^*)}\\&\quad \le \int _0^1 \left\Vert {{\mathcal {F}}}'(y_1+t(y_2-y_1)) {{\mathcal {F}}}'(0) \right\Vert _{{{\mathcal {L}}}(W_{\infty }, L^2(I,V^*))}\, dt \, \Vert y_2-y_1\Vert _{W_\infty }\\&\quad \le \int _0^1 \int _0^1 \left\Vert {{\mathcal {F}}}''(s(y_1+t(y_2-y_1)))(ty_2+(1-t)y_1)\right\Vert _{{{\mathcal {L}}}(W_{\infty }, L^2(I,V^*))}\,\\&\qquad ds dt \, \Vert y_2-y_1\Vert _{W_\infty }. \end{aligned}$$

Now the claim follows by assumption (A3). $\square$

#### Lemma 4.2

Let $\displaystyle {{\mathcal {A}}}_s$ be the generator of an exponentially stable analytic semigroup $\displaystyle e^{{{\mathcal {A}}}_s t}$ on *Y*. Let *C* denote the constant from Lemma 4.1. Then there exists a constant $M_s$ such that for all $y_0 \in Y$ and $f \in L^2(I; V^*)$ with

$$\begin{aligned} {\tilde{\gamma }} = \left\Vert y_0\right\Vert _Y + \left\Vert f\right\Vert _{L^2(I; V^*)} \le \frac{1}{4CM^2_s} \end{aligned}$$

the system

4.5 $$\begin{aligned} y_t = {{\mathcal {A}}}_s y + {{\mathcal {F}}}(y) + f, \quad y(0) = y_0 \end{aligned}$$

has a unique solution $y \in W_{\infty }$, which satisfies

$$\begin{aligned} \left\Vert y\right\Vert _{W_{\infty }} \le 2 M_s {\tilde{\gamma }}. \end{aligned}$$

With Lemma 4.1 holding, this lemma can be verified in the same manner as [6, Lemma 5, p. 6]. In the following corollary we shall use Lemma 4.2 with ${{\mathcal {A}}}_s = {{\mathcal {A}}}-BK$, and the constant corresponding to $M_s$ will be denoted by $M_K$. Further $\Vert {{\mathcal {I}}}\Vert$ denotes the norm of the embedding constant of $W_\infty$ into *C*(*I*; *Y*), $\Vert i\Vert$ is the norm of the embedding *V* into *Y*, and we recall the constant $\eta$ from (3.1).

#### Corollary 4.3

For all $\displaystyle y_0 \in Y$ with

$$\begin{aligned} \left\Vert y_0\right\Vert _Y \le \min \left\{ \frac{1}{4CM^2_K}, \frac{\eta }{2M_K\left\Vert K\right\Vert _{{{\mathcal {L}}}(Y)}\left\Vert {{\mathcal {I}}}\right\Vert } \right\} \end{aligned}$$

there exists a control $u \in {U_{ad}}$ such that the system

4.6 $$\begin{aligned} y_t = {{\mathcal {A}}}y + {{\mathcal {F}}}(y) + Bu, \quad y(0) = y_0 \end{aligned}$$

has a unique solution $y \in W_{\infty }$ satisfying

4.7 $$\begin{aligned} \left\Vert y\right\Vert _{W_{\infty }}\le & {} 2 M_K \Vert y_0\Vert _Y \quad \text { and } \quad \left\Vert u\right\Vert _{U} \le \Vert K\Vert _{{{\mathcal {L}}}(Y,\,{{\mathcal {U}}})} \Vert {{\mathcal {I}}}\Vert \Vert y\Vert _{W_\infty }\nonumber \\\le & {} 2M_K \Vert y_0\Vert _Y \Vert K\Vert _{{{\mathcal {L}}}(Y,\,{{\mathcal {U}}})}\Vert {{\mathcal {I}}}\Vert . \end{aligned}$$

#### Proof

By Assumption (A2), there exists *K* such that ${{\mathcal {A}}}- BK$ generates an exponentially stable analytic semigroup on *Y*. Taking $u = -Ky$, equation (4.6) becomes

4.8 $$\begin{aligned} y_t = ({{\mathcal {A}}}- BK) y + {{\mathcal {F}}}(y), \quad y(0) = y_0. \end{aligned}$$

Then by Lemma 4.2 with ${\tilde{\gamma }} = \Vert y_0\Vert _Y$ there exists $M_K$ such that (4.8) has a solution $y\in W_\infty$ satisfying

$$\begin{aligned} \left\Vert y\right\Vert _{W_{\infty }} \le 2 M_K \Vert y_0\Vert _Y, \end{aligned}$$

and thus the first inequality in (4.7) holds. For every $t\in I$ we have

4.9 $$\begin{aligned} \Vert u \Vert _U= & {} \Vert Ky\Vert _U \le \Vert K\Vert _{{{\mathcal {L}}}(Y,\,{{\mathcal {U}}})} \Vert y \Vert _Y \le \Vert K \Vert _{{{\mathcal {L}}}(Y,\,{{\mathcal {U}}})} \Vert {{\mathcal {I}}}\Vert \Vert y \Vert _{W_\infty }\nonumber \\\le & {} 2M_K \Vert y_0 \Vert _Y \Vert K \Vert _{{{\mathcal {L}}}(Y,\,{{\mathcal {U}}})} \Vert {{\mathcal {I}}}\Vert , \end{aligned}$$

and thus the second inequality in (4.7) holds.We still need to assert that $u \in U_{ad}$. This follows from the second smallness condition on $\left\Vert y_0\right\Vert _Y$ and (4.9). $\square$

#### Remark 4.1

In the above proof stabilization was achieved by the feedback control $u = -Ky$. For this *u* to be admissible it is needed that ${{\mathcal {U}}}_{ad}$ has nonempty interior. The upper bound $\eta$ could be allowed to be time dependent as long as it satisfies $\displaystyle \inf _{t\ge 0}|\eta (t)|>0$.

#### Corollary 4.4

Let $\displaystyle y_0 \in Y$ and let $u \in U_{ad}$ be such that the system

4.10 $$\begin{aligned} y_t = {{\mathcal {A}}}y + {{\mathcal {F}}}(y) + Bu, \quad y(0) = y_0 \end{aligned}$$

has a unique solution $y \in L^2(I;Y)$. If

$$\begin{aligned} \gamma : = \left\Vert y_0\right\Vert _Y + \left\Vert \rho y + Bu\right\Vert _{L^2(I; V^*)} \le \min \left\{ \frac{1}{4CM^2_{\rho }}, \frac{\eta }{2M_{\rho }\left\Vert K\right\Vert _{{{\mathcal {L}}}(Y)}\left\Vert {{\mathcal {I}}}\right\Vert } \right\} , \end{aligned}$$

then $y \in W_{\infty }$ and it holds that

$$\begin{aligned} \left\Vert y\right\Vert _{W_{\infty }} \le 2 M_{\rho } \gamma . \end{aligned}$$

#### Proof

Since $y \in L^2(I; Y)$, we can apply Lemma 4.2 to the equivalent system

$$\begin{aligned} y_t = ({{\mathcal {A}}}- \rho I)y + {{\mathcal {F}}}(y) + {\tilde{f}}, \end{aligned}$$

where ${\tilde{f}} = \rho y + Bu$. This proves the assertion. $\square$

#### Lemma 4.5

There exists $\delta _1 > 0$ such that for all $\displaystyle y_0 \in B_Y(\delta _1)$, problem (${\mathcal {P}}$) possesses a solution $(\bar{y}, \bar{u}) \in W_{\infty } \times U_{ad}$. Moreover, there exists a constant $M > 0$ independent of $y_0$ such that

4.11 $$\begin{aligned} \max \big \{ \left\Vert \bar{y}\right\Vert _{W_{\infty }}, \left\Vert \bar{u}\right\Vert _U \big \} \le M \left\Vert y_0\right\Vert _{Y} \end{aligned}$$

#### Proof

The proof of this lemma follows with analogous argumentation as provided in [6, Lemma 8]. Let us choose, $\delta _1 \le \min \left\{ \frac{1}{4CM^2_K}, \frac{\eta }{2M_K\left\Vert K\right\Vert _{{{\mathcal {L}}}(Y)}\left\Vert {{\mathcal {I}}}\right\Vert } \right\}$, where *C* as in Lemma  4.1 and $M_K$ denotes the constant from the Corollary 4.3. We obtain that for each $y_0 \in B_Y(\delta _1)$, there exists a control $u \in U_{ad}$ with associated state *y* satisfying

4.12 $$\begin{aligned} \max \big \{ \left\Vert u\right\Vert _{U}, \left\Vert y\right\Vert _{W_{\infty }} \big \} \le {{\tilde{M}}} \left\Vert y_0\right\Vert _Y, \end{aligned}$$

where $\displaystyle {{\tilde{M}}} = 2M_K \text { max } \big (1, \left\Vert i\right\Vert \left\Vert K\right\Vert _{{{\mathcal {L}}}(Y,\,{{\mathcal {U}}})} \big )$. We can thus consider a minimizing sequence $\displaystyle (y_n, u_n)_{n \in {\mathbb {N}}} \in W_\infty \times U_{ad}$ with $\displaystyle J(y_n, u_n) \le \frac{1}{2}M^2 \left\Vert y_0\right\Vert ^2_Y(1 + \alpha )$. For all $n \in {\mathbb {N}}$ that

4.13 $$\begin{aligned} \left\Vert y_n\right\Vert _{L^2(I;Y)} \le {{\tilde{M}}} \left\Vert y_0\right\Vert _Y \sqrt{1 + \alpha } \quad \text { and } \quad \left\Vert u_n\right\Vert _{L^2(I;\,{{\mathcal {U}}})} \le {{\tilde{M}}} \left\Vert y_0\right\Vert _Y \sqrt{\frac{1 + \alpha }{\alpha }}.\nonumber \\ \end{aligned}$$

We set $\eta (\alpha ,{{\tilde{M}}}) = \Big [ 1 + {{\tilde{M}}} \Vert i\Vert \ \sqrt{(1 + \alpha )} \Big ( \rho + \frac{\left\Vert B\right\Vert _{{{\mathcal {L}}}({{\mathcal {U}}}, Y)}}{\sqrt{\alpha }} \Big ) \Big ]$. Then we have $\left\Vert y_0\right\Vert + \left\Vert \rho y_n + B u_n \right\Vert _{L^2(I;V^*)} \le \eta (\alpha ,{{\tilde{M}}}) \left\Vert y_0\right\Vert _Y$. After further reduction of $\delta _1$, we obtain with $M_{\rho }$ from Corollary 4.4:

$$\begin{aligned} \left\Vert y_0\right\Vert + \left\Vert \rho y_n + B u_n \right\Vert _{L^2(I;V^*)} \le \frac{1}{4CM^2_{\rho }}. \end{aligned}$$

It follows from this corollary that the sequence $\{y_n\}_{n \in {\mathbb {N}}}$ is bounded in $W_{\infty }$ with

4.14 $$\begin{aligned} \sup _{n \in {\mathbb {N}}} \left\Vert y_n\right\Vert _{W_{\infty }} \le 2 M_\rho (1+\eta (\alpha , {{\tilde{M}}})) \left\Vert y_0\right\Vert _{Y}. \end{aligned}$$

Extracting if necessary a subsequence, there exists $\displaystyle (\bar{y}, \bar{u}) \in W_{\infty } \times U$ such that $\displaystyle ({y}_n, {u}_n) \rightharpoonup (\bar{y}, \bar{u}) \in W_{\infty } \times U$, and $(\bar{y}, \bar{u})$ satisfies (4.12).

Let us prove that $(\bar{y}, \bar{u})$ is feasible and optimal. Since $U_{ad}$ is weakly sequentially closed and $u_n \in U_{ad}$, we find $\bar{u} \in U_{ad}$. For each fixed $T > 0$ and arbitrary $z \in L^{\infty }(0;T;{{\mathcal {H}}}) \subset L^2(0,T;V)$, see (A4), we have for all $\displaystyle n \in {\mathbb {N}}$ that

4.15 $$\begin{aligned} \int _{0}^{T} \langle {\dot{y}}_n(t), z(t) \rangle _{V^*,V} dt = \int _{0}^{T} \langle {{\mathcal {A}}}y_n (t) + {{\mathcal {F}}}(y_n(t)) + Bu_n(t), z(t) \rangle _{V^*,V} dt.\nonumber \\ \end{aligned}$$

Since ${\dot{y}}_n \rightharpoonup {\dot{y}}$ in $L^2(0,T; V^*)$, we can pass to the limit in the l.h.s. of the above equality. Moreover, since ${{\mathcal {A}}}y_n \rightharpoonup {{\mathcal {A}}}y$ in $L^2(0,T; V^*)$,

$$\begin{aligned} \int _{0}^{T} \langle {{\mathcal {A}}}y_n (t), z(t) \rangle _{V^*,V} dt \xrightarrow [n \rightarrow \infty ]{} \int _{0}^{T} \langle {{\mathcal {A}}}\bar{y}(t), z(t) \rangle _{V^*,V} dt. \end{aligned}$$

Analogously, we obtain that

$$\begin{aligned} \int _{0}^{T} \langle B u_n (t), z(t) \rangle _{V^*,V} dt \xrightarrow [n \rightarrow \infty ]{} \int _{0}^{T} \langle B \bar{u}(t), z(t) \rangle _{V^*,V} dt. \end{aligned}$$

If moreover $z \in L^{\infty }(0,T;{{\mathcal {H}}}) \subset L^2(0,T;V)$, we use (A4) to assert

$$\begin{aligned} \int _{0}^{T} \langle {{\mathcal {F}}}(y_n(t)) - {{\mathcal {F}}}(\bar{y}(t)), z(t) \rangle _{V^*,V} dt =\int _{0}^{T} \langle {{\mathcal {F}}}(y_n(t)) - {{\mathcal {F}}}(\bar{y}(t)), z(t) \rangle _{{{\mathcal {H}}}^*,{{\mathcal {H}}}} dt \xrightarrow [n \rightarrow \infty ]{} 0 . \end{aligned}$$

Thus we have for all $z \in L^{\infty }(0,T;{{\mathcal {H}}})$

4.16 $$\begin{aligned} \int _{0}^{T} \langle {\dot{y}}(t) - {{\mathcal {A}}}y(t) - Bu(t), z(t) \rangle _{V^*,V} dt = \int _{0}^{T} \langle {{\mathcal {F}}}(y(t)), z(t) \rangle _{V^*,V} dt. \end{aligned}$$

Since $\displaystyle {\dot{y}} - {{\mathcal {A}}}y - Bu \in L^2(0,T;V^*)$ and $L^{\infty }(0,T;{{\mathcal {H}}})$ is dense in $L^2(0,T;V)$ we conclude that (4.16) holds for all $z \in L^2(0,T;V)$ and $T > 0$. This yields $e(\bar{y},\bar{u}) = (0,0)$, and thus $(\bar{y},\bar{u})$ is feasible. By weak lower semicontinuity of norms it follows that $\displaystyle J(\bar{y},\bar{u}) \le \liminf _{n \rightarrow \infty } J(\bar{y}_n,\bar{u}_n)$, which proves the optimality of $(\bar{y},\bar{u})$, and (4.11) follows from (4.13). $\square$

For the derivation of the optimality system for (${\mathcal {P}}$), we need the following lemma which is taken from [5, Lemma 2.5].

#### Lemma 4.6

Let $\displaystyle G \in {{\mathcal {L}}}(W_{\infty },L^2(I;V^*))$ such that $\displaystyle \left\Vert G\right\Vert < \frac{1}{M_K}$, where $\left\Vert G\right\Vert$ denotes the operator norm of *G*. Then for all $\displaystyle f \in L^2(I;V^*)$ and $y_0 \in Y$, there exists a unique solution to the problem:

$$\begin{aligned} y_t = ({{\mathcal {A}}}- BK)y(t) + (Gy)(t) + f(t), \quad y = y_0. \end{aligned}$$

Moreover,

$$\begin{aligned} \left\Vert y\right\Vert _{W_{\infty }} \le \frac{M_K}{1 - {M_K \Vert G\Vert }} \left( \left\Vert f\right\Vert _{L^2(I;V^*)} + \left\Vert y_0\right\Vert _Y \right) . \end{aligned}$$

We close this section by deriving the optimality conditions for (${\mathcal {P}}$).

#### Proposition 1

Let the assumptions (A1) - (A4) hold. Then there exists $\delta _2 \in (0,\delta _1]$ such that each local solution $(\bar{y}, \bar{u})$ with $y_0 \in B_Y(\delta _2)$ is a regular point, i.e. (2.3) is satisfied, and there exists an adjoint state $({\bar{p}}, {\bar{p}}_1) \in L^2(I;V) \times Y$ satisfying

4.17 $$\begin{aligned}&\langle v_t - {{\mathcal {A}}}v - {{\mathcal {F}}}'(\bar{y})v, {\bar{p}} \rangle _{L^2(I;V^*), L^2(I;V)} + ( v(0), {\bar{p}}_1 )_Y + ( {\bar{y}}, {\bar{p}} )_{L^2(I;V)} = 0, \quad \nonumber \\&\qquad \text {for all } v \in W_{\infty }, \end{aligned}$$

4.18 $$\begin{aligned}&\langle \alpha \bar{u} - B^* {\bar{p}}, u - \bar{u} \rangle _{U} \ge 0, \quad \text {for all } u \in U_{ad}. \end{aligned}$$

If the assumption (A5) is satisfied, then

$$\begin{aligned} - {\bar{p}}_t - {{\mathcal {A}}}^* {\bar{p}} - {{\mathcal {F}}}'(\bar{y})^* {\bar{p}} = -\bar{y} \quad \text { in } L^2(I;V^*), \end{aligned}$$

and hence ${\bar{p}} \in W_{\infty }$ and

4.19 $$\begin{aligned} \lim _{t \rightarrow \infty } {\bar{p}}(t) = 0. \end{aligned}$$

Moreover, there exists $\widetilde{M} > 0$, independent of $y_0 \in B_Y(\delta _2)$, such that

4.20 $$\begin{aligned} \left\Vert {\bar{p}}\right\Vert _{W_{\infty }} \le \widetilde{M} \left\Vert y_0\right\Vert _Y, \text { and } u \in C(\overline{I}, {{\mathcal {U}}}). \end{aligned}$$

#### Proof

To verify the regular point condition, we evaluate *e* defined in (3.6) at $(\bar{y}, \bar{u}, y_0)$. To check the claim on the range of $e'(\bar{y}, \bar{u}, y_0)$ we consider for arbitrary $\displaystyle (r,s) \in L^2(I,V^*) \times Y$ the equation

4.21 $$\begin{aligned} z_t - {{\mathcal {A}}}z - {{\mathcal {F}}}'(\bar{y})z - B(w - \bar{u}) = r, \ z(0) = s, \end{aligned}$$

for unknowns $(z,w) \in W_{\infty } \times {U_{ad}}$. By taking $w = -Kz \in U$ we obtain

$$\begin{aligned} z_t - ({{\mathcal {A}}}- BK)z - {{\mathcal {F}}}'(\bar{y})z + B \bar{u} = r, \ z(0) = s. \end{aligned}$$

We apply Lemma 4.6 to this equation with $\displaystyle G = - {{\mathcal {F}}}'(\bar{y})$ and $f = r - B \bar{u}$. By Lemma 4.5 and (3.3) in Remark 3.1 there exists $\delta _2 \in (0,\delta _1]$ such that $\Vert {{\mathcal {F}}}'(\bar{y})\Vert _{{{\mathcal {L}}}(W_{\infty },L^2(I;V^*))} \le \frac{1}{2} M_K$. Consequently by Lemma 4.6 there exists $\widetilde{M}$ such that

4.22 $$\begin{aligned} \left\Vert z\right\Vert _{W_{\infty }}&\le \widetilde{M} \big ( \left\Vert r\right\Vert _{L^2(I; V^*)} + \left\Vert s\right\Vert _{Y} + \left\Vert B\right\Vert _{ {{\mathcal {L}}}({{\mathcal {U}}}, Y)} \left\Vert \bar{u}\right\Vert _U\big ) \nonumber \\&\le \widetilde{M} \big ( \left\Vert r\right\Vert _{L^2(I; V^*)} + \left\Vert s\right\Vert _{Y} + \left\Vert B\right\Vert _{{{\mathcal {L}}}({{\mathcal {U}}}, Y)} M \left\Vert y_0\right\Vert _Y \big ) , \end{aligned}$$

with *M* as in (4.11). We shall need to check whether $w = -Kz$ is feasible, which will be the case if $w(t) \le \eta$ for a.e. $t \in I$. Indeed we have

$$\begin{aligned} \left\Vert w(t)\right\Vert _Y&\le \left\Vert K\right\Vert _{{{\mathcal {L}}}(Y,\,{{\mathcal {U}}})} \left\Vert z(t)\right\Vert _{Y}\\&\le \left\Vert K\right\Vert _{{{\mathcal {L}}}(Y,\,{{\mathcal {U}}})} \left\Vert {{\mathcal {I}}}\right\Vert \widetilde{M} \big ( \left\Vert r\right\Vert _{L^2(I; V^*)} + \left\Vert s\right\Vert _{Y} + \left\Vert B\right\Vert _{{{\mathcal {L}}}({{\mathcal {U}}}, Y)} M \left\Vert y_0\right\Vert _Y \big ). \end{aligned}$$

Consequently, possibly after further reducing $\delta _2$, and choosing $\tilde{\delta } > 0$ sufficiently small we have

4.23 $$\begin{aligned} \left\Vert w\right\Vert _{L^{\infty }(I;Y)} \le \eta \text { for all } \displaystyle y_0 \in B_Y(\delta _2) \text { and all } (r,s) \text { satisfying } \left\Vert (r,s)\right\Vert _{L^2(I;V^*) \times Y} \le \tilde{\delta }. \end{aligned}$$

Consequently the regular point condition is satisfied. Hence there exists a multiplier $\displaystyle \lambda = (p,p_1) \in L^2(I;V) \times Y$ satisfying,

4.24 $$\begin{aligned}&\langle {{\mathcal {L}}}_y (\bar{y}, \bar{u}, y_0, {\bar{p}}, \bar{p}_1), v \rangle _{L^2(I;V^*), L^2(I;V)} = 0, \quad \nonumber \\&\quad \langle {{\mathcal {L}}}_u (\bar{y}, \bar{u}, y_0, {\bar{p}}, {\bar{p}}_1), u - \bar{u} \rangle _{U} \ge 0, \ \forall u \in U_{ad}\end{aligned}$$

where

$$\begin{aligned} {{\mathcal {L}}}(y, u, y_0, p, p_1) = J(y,u) + \int _0^{\infty } \langle p, y_t - {{\mathcal {A}}}y - {{\mathcal {F}}}(y) - Bu \rangle _{V, V^*}dt + \langle p_1, y(0) - y_0 \rangle _Y. \end{aligned}$$

This implies that (4.17) holds.

Now, if we impose the additional assumption (A5), we have ${{\mathcal {F}}}'(\bar{y})^* {\bar{p}} \in L^2(I;V^*)$. Thus $- {{\mathcal {A}}}^* {\bar{p}} - {{\mathcal {F}}}'(\bar{y})^* {\bar{p}} + \bar{y} \in L^2(I;V^*)$ and the previous identity implies that ${\bar{p}} \in W_{\infty }$. Thus we derive

$$\begin{aligned} - {\bar{p}}_t - {{\mathcal {A}}}^* {\bar{p}} - {{\mathcal {F}}}'(\bar{y})^* {\bar{p}} = -\bar{y} \ \text {in } L^2(I;V^*) \text { and } \lim _{t \rightarrow \infty } \bar{p}(t) = 0, \end{aligned}$$

and (4.17)-(4.19). Testing the first identity in (4.24) with $v\in L^2(I;V)$ we also have ${\bar{p}}_1 = {\bar{p}}(0)$, which is well-defined since ${\bar{p}} \in W_{\infty } \subset C(I;Y)$. The second identity in (4.24) gives (4.18). It remains to estimate $\displaystyle {\bar{p}} \in W_\infty$.

Let $\displaystyle r \in L^2(I;V^*)$ with $\displaystyle \left\Vert r\right\Vert _{L^2(I;V^*)} \le \tilde{\delta }$ and consider

4.25 $$\begin{aligned} z_t - {{\mathcal {A}}}z - {{\mathcal {F}}}'(\bar{y})z - B (w-\bar{u}) = -r, \ z(0) = 0. \end{aligned}$$

Arguing as in (4.21)-(4.22) there exists a solution to (4.25) with $w = - Kz$ such that

4.26 $$\begin{aligned} \left\Vert z\right\Vert _{W_{\infty }} \le \widetilde{M} \big ( \tilde{\delta } + \left\Vert B\right\Vert _{{{\mathcal {L}}}({{\mathcal {U}}}, Y)} M \left\Vert y_0\right\Vert _Y \big ) \le \widetilde{M} \big ( \tilde{\delta } + \left\Vert B\right\Vert _{{{\mathcal {L}}}({{\mathcal {U}}}, Y)} M \delta _2 \big ) =: C_1.\nonumber \\ \end{aligned}$$

From (4.23) we have that $\displaystyle \left\Vert w\right\Vert _{L^{\infty }(I,\,{{\mathcal {U}}})} \le \eta$. Let us now observe that

$$\begin{aligned}&\langle {\bar{p}}, r \rangle _{L^2(I,V), L^2(I,V^*)} \\&\quad = \langle {\bar{p}}, -z_t + {{\mathcal {A}}}z + {{\mathcal {F}}}'(\bar{y})z \rangle _{L^2(I,V), L^2(I,V^*)} + \langle {\bar{p}}, B(w-\bar{u}) \rangle _{L^2(I;Y)}\\&\quad = \langle {\bar{p}}_t + {{\mathcal {A}}}^* {\bar{p}} + {{\mathcal {F}}}'(\bar{y})^* {\bar{p}}, z \rangle + \langle B^* {\bar{p}}, w - \bar{u} \rangle _U, \end{aligned}$$

where we have used that $z(0) = 0$ and $\displaystyle \lim _{t \rightarrow \infty } {\bar{p}}(t) = 0$, since ${\bar{p}} \in W_\infty$. We next estimate using (4.17), (4.19) and (4.26)

$$\begin{aligned} \langle {\bar{p}}, r \rangle _{L^2(I,V), L^2(I,V^*)}&\le \left\Vert \bar{y}\right\Vert _{L^2(I,V^*)} \left\Vert z\right\Vert _{L^2(I,V)} + \alpha \langle \bar{u}, w - \bar{u} \rangle _U\\&\le \left( \left\Vert \bar{y}\right\Vert _{L^2(I,V^*)} + \alpha \left\Vert \bar{u}\right\Vert _U \right) \left( C_1 + \eta + \left\Vert \bar{u}\right\Vert _U \right) . \end{aligned}$$

By (4.11), this implies the existence of a constant $C_2$ such that

$$\begin{aligned} \sup _{\left\Vert r\right\Vert _{L^2(I,V^*)} \le {\tilde{\delta }}} \langle \bar{p}, r \rangle _{L^2(I,V), L^2(I,V^*)} \le C_2 \left\Vert y_0\right\Vert _Y \end{aligned}$$

and thus

4.27 $$\begin{aligned} \left\Vert {\bar{p}}\right\Vert _{L^2(I,V)} \le \frac{C_2}{{\tilde{\delta }}} \left\Vert y_0\right\Vert _Y, \quad \text {for all } y_0 \in B_Y(\delta _2). \end{aligned}$$

Now we estimate, again using (A5)

$$\begin{aligned} \left\Vert {\bar{p}}_t\right\Vert _{L^2(I;V^*)}&\le \left\Vert {{\mathcal {A}}}^* {\bar{p}} + {{\mathcal {F}}}'(\bar{y})^* {\bar{p}} - \bar{y}\right\Vert _{L^2(I;V^*)}\\&\le C_3\left\Vert \bar{p}\right\Vert _{L^2(I,V)} + C_4 \left\Vert {\bar{p}}\right\Vert _{L^2(I,V)} + \left\Vert \bar{y}\right\Vert _{L^2(I;V^*)}. \end{aligned}$$

By (4.11) and (4.27) we obtain $\left\Vert {\bar{p}}_t\right\Vert _{L^2(I;V^*)} \le C_5 \left\Vert y_0\right\Vert _Y.$ Combining this estimate with (4.27) yields (4.20). Finally, by (4.18) we find $\displaystyle {\bar{u}}(t) = {\mathbb {P}}_{{{\mathcal {U}}}_{ad}} \left( \frac{1}{\alpha } B^* \bar{p}(t) \right)$. Since ${\bar{p}} \in C({\bar{I}}; Y)$ and $B^* \in {{\mathcal {L}}}(Y,U)$ this implies that $u \in C({\bar{I}}; {{\mathcal {U}}})$. $\square$

### Verification of (H1)–(H6)

In this section we specialize the previously proved abstract results in Sect. 2 to the semilinear parabolic setting. We start with the following lemma which shows that assumptions A imply (H1)-(H6).

#### Lemma 4.7

Consider problem (${\mathcal {P}}$) with assumptions (A1)-(A4) holding. Then (H1)–(H4), (H6) are satisfied for (${\mathcal {P}}$) uniformly for all $y_0 \in B_Y(\widetilde{\delta }_2)$ for some $\displaystyle \widetilde{\delta }_2 \in (0, \delta _2]$. If moreover, (A5) holds, then (H5) holds as well.

#### Proof

Throughout $y_0\! \in \! B_Y(\delta _2)$, $({\bar{y}}, {\bar{u}})$ denotes a local solution to (${\mathcal {P}}$), and $(p,p_1) \in L^2(I;V^*) \times Y$ the associated Lagrange multiplier.

(i) Verification of (H1): The initial condition $y_0$ is our nominal reference parameter *q*. Lemma 4.5 guarantees the existence of a local solution $(\bar{y}, \bar{u}) \sim x_0$ to (${\mathcal {P}}$)$\sim$ ($P_{q_0}$). Clearly *f* defined in (3.5) satisfies the required regularity assumptions. Moreover *e* satisfies the regularity assumptions as a consequence of (A3).
(ii) Verification of (H2): Proposition 1 implies that $(\bar{y}, \bar{u})$ is a regular point.
(iii) Verification of (H3): The second derivative of *e* is given by
  4.28 $$\begin{aligned}&e''(\bar{y}, \bar{u}, y_0)((v_1,w_1), (v_2,w_2))\nonumber \\&\quad = \begin{pmatrix}{{\mathcal {F}}}''(\bar{y}) (v_1, v_2) \\ 0 \end{pmatrix}, \quad \forall \ v_1, v_2 \in W_{\infty }, \ \forall w_1, w_2 \in U. \end{aligned}$$
  For the second derivative of ${{\mathcal {L}}}$ w.r.t. (*y*, *u*), we find
  4.29 $$\begin{aligned}&{{\mathcal {L}}}''(\bar{y},\bar{u},y_0, {\bar{p}}, {\bar{p}}_1)((v_1,w_1),(v_2,w_2))\nonumber \\&\quad = \int _{0}^{\infty } ( v_1, v_2 )_Y dt + \alpha \int _{0}^{\infty } ( w_1, w_2 )_Y dt \nonumber \\&\qquad + \int _{0}^{\infty } \langle \bar{p}, {{\mathcal {F}}}''(\bar{y})(v_1, v_2) \rangle _{V,V^*} dt. \end{aligned}$$
  By (A3) for ${{\mathcal {F}}}''$ and Lemma 4.5 , there exists $\widetilde{M}_1$ such that
  4.30 $$\begin{aligned} \int _{0}^{\infty } \langle {\bar{p}}, {{\mathcal {F}}}''(\bar{y})(v, v) \rangle _{V,V^*} dt \le \widetilde{M}_1 \Vert {\bar{p}} \Vert _{L^2(I;V)} \left\Vert v\right\Vert ^2_{W_{\infty }}, \quad \forall \ v\in W_\infty ,\qquad \quad \end{aligned}$$
  for each solution $(\bar{y}, \bar{u})$ of (${\mathcal {P}}$) with $y_0 \in B_Y(\delta _2)$. Then we obtain
  4.31 $$\begin{aligned}&{{\mathcal {L}}}''(\bar{y}, \bar{u}, y_0, {\bar{p}}, {\bar{p}}_1)((v,w),(v,w)) \nonumber \\&\quad \ge \int _{0}^{\infty } \left\Vert v\right\Vert _Y^2 dt\nonumber \\&\qquad + \alpha \int _{0}^{\infty } \left\Vert w\right\Vert ^2_{{{\mathcal {U}}}} dt - \widetilde{M}_1 \Vert {\bar{p}} \Vert _{L^2(I;V)} \left\Vert v\right\Vert ^2_{W_{\infty }}. \end{aligned}$$
  Now let $0\ne (v,w) \in \ker E \subset W_{\infty } \times U_{ad}$, where *E* as defined in (3.7) is evaluated at $(\bar{y},{\bar{u}})$. Then,
  $$\begin{aligned} v_t - {{\mathcal {A}}}v - {{\mathcal {F}}}'(\bar{y})v - Bw = 0, \quad v(0) = 0. \end{aligned}$$
  Next choose $\rho > 0$, such that the semigroup generated by $({{\mathcal {A}}}- \rho I)$ is exponentially stable. This is possible due to (A1). We equivalently write the system in the previous equation as,
  $$\begin{aligned} v_t - ({{\mathcal {A}}}- \rho I) v - {{\mathcal {F}}}'(\bar{y})v - \rho v - Bw = 0, \quad v(0) = 0. \end{aligned}$$
  Now, we invoke Lemma 4.6 with $\mathcal{A}-BK$ replaced by ${{\mathcal {A}}}- \rho I$, $\displaystyle G = {{\mathcal {F}}}'(\bar{y})$, and $f(t)=\rho v(t) + B w(t)$, and the role of the constant $M_K$ will now be assumed by a parameter $M_\rho$. By selecting $\widetilde{\delta }_2 \in (0, \delta _2]$ such that $\left\Vert \bar{y}\right\Vert _{W_{\infty }}$ sufficiently small, we can guarantee that $\displaystyle \left\Vert {{\mathcal {F}}}'(\bar{y})\right\Vert _{{{\mathcal {L}}}(W_{\infty }; L^2(I;V^*))} \le {}^{1}\!/_{2 M_\rho }$, see (4.11) and (3.3) in Remark 3.1. Then the following estimate holds,
  $$\begin{aligned} \left\Vert v\right\Vert _{W_{\infty }} \le 2{M_\rho } \left\Vert v + Bw\right\Vert _{L^2(I; V^*)}. \end{aligned}$$
  This implies that
  4.32 $$\begin{aligned} \left\Vert v\right\Vert ^2_{W_{\infty }} \le \widetilde{M}_2 ( \left\Vert v\right\Vert ^2_{L^2(I; Y)} + \left\Vert w\right\Vert ^2_{L^2(I; Y)} ). \end{aligned}$$
  for a constant $\widetilde{M}_2$ depending on $M_\rho , \Vert B\Vert$, and the embedding of *Y* into $V^*$. These preliminaries allow the following lower bound on ${{\mathcal {L}}}''$:
  4.33 $$\begin{aligned}&{{\mathcal {L}}}''(\bar{y}, \bar{u}, y_0, {\bar{p}}, {\bar{p}}_1)((v,w),(v,w))\nonumber \\&\quad \ge \int _{0}^{\infty } \left\Vert v\right\Vert _Y^2 dt + \alpha \int _{0}^{\infty } \left\Vert w\right\Vert ^2_Y dt - \widetilde{M}_1 \Vert {\bar{p}}\Vert _{L^2(I;V)} \left\Vert v\right\Vert ^2_{W_{\infty }} \nonumber \\&\qquad \text {by ~ (4.32) ~} \ge \int _{0}^{\infty } \left\Vert v\right\Vert _Y^2 \nonumber \\&\quad + \alpha \int _{0}^{\infty } \left\Vert w\right\Vert ^2_Y - \widetilde{M}_1 \widetilde{M}_2 \Vert {\bar{p}} \Vert _{L^2(I;V)} \left[ \left\Vert v\right\Vert ^2_{L^2(I; Y)} + \left\Vert w\right\Vert ^2_{L^2(I; Y)} \right] \nonumber \\&\quad = \left( 1 - \widetilde{M}_1 \widetilde{M}_2 \right. \left. \Vert {\bar{p}} \Vert _{L^2(I;V)} \right) \left\Vert v\right\Vert ^2_{L^2(I;Y)} + \left( \alpha - \widetilde{M}_1 \widetilde{M}_2 \Vert {\bar{p}} \Vert _{L^2(I;V)} \right) \left\Vert w\right\Vert ^2_{L^2(I;Y)} \nonumber \\&\quad \ge \tilde{\gamma } \left[ \left\Vert v\right\Vert ^2_{L^2(I;Y)} + \left\Vert w\right\Vert ^2_{L^2(I;Y)} \right] \end{aligned}$$
  where $\displaystyle \tilde{\gamma } = \min \left\{ 1 - \widetilde{M}_1 \widetilde{M}_2 \Vert {\bar{p}} \Vert _{L^2(I;V)} , \alpha - \widetilde{M}_1 \widetilde{M}_2 \Vert {\bar{p}} \Vert _{L^2(I;V)} \right\}$. By possible further reduction of $\tilde{\delta }_2$ it can be guaranteed that ${\tilde{\gamma }} >0$, see (4.27). Then by (4.32), we obtain,
  $$\begin{aligned}&{{\mathcal {L}}}''(\bar{y}, \bar{u}, y_0, {\bar{p}}, {\bar{p}}_1)((v,w),(v,w))\\&\quad \ge \frac{\tilde{\gamma }}{2} \left[ \left\Vert v\right\Vert ^2_{L^2(I;Y)} + \left\Vert w\right\Vert ^2_{L^2(I;Y)} \right] + \frac{\tilde{\gamma }}{2 \widetilde{M}_2} \left\Vert v\right\Vert ^2_{W_{\infty }},\\&\quad \ge \frac{\tilde{\gamma }}{2 \widetilde{M}_2} \left\Vert v\right\Vert ^2_{W_{\infty }} + \frac{\tilde{\gamma }}{2} \left\Vert w\right\Vert ^2_{L^2(I;Y)}. \end{aligned}$$
  By selecting $\displaystyle {\bar{\gamma }} = \min \left\{ \frac{\tilde{\gamma }}{2 \widetilde{M}_2}, \frac{\tilde{\gamma }}{2} \right\}$, we obtain the positive definiteness of ${{\mathcal {L}}}''$, i.e.
  4.34 $$\begin{aligned}&{{\mathcal {L}}}''(\bar{y}, \bar{u}, y_0, {\bar{p}}, {\bar{p}}_1)((v,w),(v,w))\nonumber \\&\quad \ge {\bar{\gamma }} \left\Vert (v,w)\right\Vert ^2_{W_{\infty } \times U}, \ y_0 \in B_Y(\widetilde{\delta }_2), \ (v,w) \in \text {ker } E. \end{aligned}$$
  Thus (H3) is satisfied.
(iv) Verification of (H4): It can easily be checked that $f'(y,u)$ can be extended to an element in $\underline{X^*} = L^2(I;V^*) \times (U \cap C({\bar{I}};{{\mathcal {U}}}))$ for each $(y,u) \in X = W_\infty \times U$. We refer to Remark 3.2 to show that the restriction of $e'(y,u,y_0)^*$ to $\underline{W^*}$ satisfies $\underline{e'(y,u,y_0)^*} \in {{\mathcal {L}}}(\underline{W^*}, \underline{X^*}) = {{\mathcal {L}}}(W_\infty \times Y,L^2(I;V^*)\times (U \cap C({\bar{I}};{{\mathcal {U}}})))$.
(v) Verification of (H6): This is trivially satisfied. Thus we have proved that assumptions (A1)-(A4) imply (H1)-(H4), and (H6) for all $\displaystyle y_0 \in B_Y(\widetilde{\delta }_2)$.
(vi) Verification of (H5): Here we use (A5) and have $(p,p_1) = (p,p(0)) \in \widetilde{W}_{\infty }$. Observe that $\underline{{{\mathcal {L}}}'}:W_{\infty } \times (U \cap C({\bar{I}};{{\mathcal {U}}})) \times Y \times W_\infty \rightarrow L^2(I;V^*) \times (U \cap C({\bar{I}};{{\mathcal {U}}}))$ evaluated at $({\bar{y}}, {\bar{u}}, y_0, p)$ is given by
  $$\begin{aligned} \underline{{{\mathcal {L}}}'}({\bar{y}}, {\bar{u}}, y_0, {\bar{p}}) = \begin{pmatrix}{\bar{y}} + \underline{e'( {\bar{y}}, {\bar{u}}, y_0)^*} {\bar{p}} \\ \alpha {\bar{u}} - B^* {\bar{p}} \end{pmatrix}= \begin{pmatrix}{\bar{y}} - {\bar{p}}_t - {{\mathcal {A}}}^* {\bar{p}} - {{\mathcal {F}}}'({\bar{y}})^* {\bar{p}} \\ \alpha {\bar{u}} - B^* {\bar{p}} \end{pmatrix}. \end{aligned}$$
  Further for $(v.w)\in W_\infty \times U$ we have
  $$\begin{aligned}&(\underline{{{\mathcal {L}}}'})'({\bar{y}}, {\bar{u}}, y_0, {\bar{p}}) (v,w) \\&\quad = \begin{pmatrix}v - [{{\mathcal {F}}}'({\bar{y}})^*]'({\bar{p}},v) \\ \alpha w \end{pmatrix}\in L^2(I;V^*)\times (U \cap C({\bar{I}};{{\mathcal {U}}})). \end{aligned}$$
  By (A3) and Remark 3.2 we have that $\underline{{{\mathcal {L}}}'}$ and $(\underline{{{\mathcal {L}}}'})'$ are continuous as mappings from $W_{\infty } \times (U \cap C({\bar{I}};{{\mathcal {U}}})) \times Y \times W_\infty$ to $L^2(I;V^*) \times (U \cap C({\bar{I}};{{\mathcal {U}}}))$, respectively to ${{\mathcal {L}}}(W_\infty \times (U \cap C({\bar{I}};{{\mathcal {U}}})); L^2(I;V^*) \times (U \cap C({\bar{I}};{{\mathcal {U}}})))$.

This proves the lemma. $\square$

#### Remark 4.2

Let us summarize our findings so far. There exists ${\tilde{\delta }}_2$ such that for each $y_0 \in B_Y({\tilde{\delta }}_2)$ problem (${\mathcal {P}}$) possesses a solution $({\bar{y}}, {\bar{u}}) \in W_{\infty } \times (U \cap C({\bar{I}};{{\mathcal {U}}}))$, with an adjoint ${\bar{p}} \in \widetilde{W}_{\infty }$. Further (A1)-(A5) imply (H1)-(H6) for (${\mathcal {P}}$) with $y_0 \in B_Y({\tilde{\delta }}_2)$. As a consequence for each $y_0 \in B_Y(\tilde{\delta }_2)$ and each associated local solution $({\bar{y}}, {\bar{u}})$ there exists a neighborhood ${{\hat{V}}}$ of the origin in ${{\mathcal {Y}}}:= L^2(I;V^*) \times (U \cap C({\bar{I}};{{\mathcal {U}}})) \times L^2(I;V^*) \times Y$ such that for each $\varvec{\beta }\in {{\hat{V}}}$ there exists a unique solution $\displaystyle \left( y_{(\varvec{\beta })}, u_{(\varvec{\beta })}, p_{(\varvec{\beta })}, {p_1}_{(\varvec{\beta })} \right) \in W_{\infty } \times U \times L^2(I;V) \times Y$ to ${{\mathcal {T}}}(y,u,p,p_1) = \varvec{\beta }$, see step (ii) of the proof of Theorem  2.1. – To verify the remaining assumption (H7) we need to argue that $\left( u_{(\varvec{\beta })}, p_{(\varvec{\beta })} \right) \in (U \cap C(\bar{I};{{\mathcal {U}}})) \times W_{\infty }$ and that $\displaystyle \varvec{\beta }\mapsto \left( y_{(\varvec{\beta })}, u_{(\varvec{\beta })}, p_{(\varvec{\beta })} \right)$ is Lipschitz continuous from ${{\hat{V}}}\subset {\mathcal {Y}}$ to $W_{\infty } \times (U \cap C({\bar{I}};{{\mathcal {U}}})) \times W_{\infty }$ .

#### Remark 4.3

Here we remark on the smallness assumption on $y_0$ expressed by $\delta _2$, respectively ${\tilde{\delta }}_2$. The condition $y_0 \in B_Y(\delta _2)$ guarantees the well-posedness of (${\mathcal {P}}$), existence and boundedness of adjoint states as expressed in Proposition 1. The additional condition $y_0 \in B_Y({\tilde{\delta }}_2)$ implies that the second order optimality condition (H3) is satisfied, for each local solution associated to an initial condition $y_0\in B_Y(\delta _2)$. In the following we formulate the results for all $y_0\in B_Y({\tilde{\delta }}_2)$. Alternatively we could narrow down the claims to neighborhoods of single local solutions $({\bar{y}}, {\bar{u}})$ with $y_0\in B_Y(\delta _2)$ and additionally assuming that the second order condition is satisfies at $({\bar{y}}, {\bar{u}})$. Concerning the second order condition itself, in some publications, see e.g. [13], it is required to hold only for elements $x=(y,u) \in \text {ker} E$ and $u = u_1-u_2$, with $u_1, u_2$ in $U_{ad}$. By a scaling argument it can easily be seen that this condition is equivalent to the one we use.

### Verification of (H7) and Lipschitz Stability of the Linearized Problem

Throughout the remainder, we assume that (A1)-(A5) are satisfied and that $y_0 \in B_Y({\tilde{\delta }}_2)$ so that Proposition 1 and Lemma 4.7 are applicable. In the following, the triple (*y*, *u*, *p*) refers to the solution ${{\mathcal {T}}}(y,u,p,p_1) = \varvec{\beta }$. Throughout without loss of generality, we also assume that ${{\hat{V}}}$ is bounded.

#### Lemma 4.8

Let assumptions (A) hold and let $({\bar{y}}, \bar{u})$, and ${\bar{p}}$ denote a local solution and associated adjoint state to (${\mathcal {P}}$) corresponding to an initial datum $y_0 \in B_Y({\tilde{\delta }}_2)$. Then the mapping $\varvec{\beta }\mapsto p_{(\varvec{\beta })}$ is continuous from ${{\hat{V}}}$ to $W_{\infty }$.

#### Proof

${\underline{\mathrm{Step\,1}:}}$ For $\varvec{\beta } \in {{\hat{V}}}$, with ${{\hat{V}}}$ as in Remark 4.2, let $\displaystyle (y_{(\varvec{\beta })}, u_{(\varvec{\beta })}, p_{(\varvec{\beta })}, {p_1}_{(\varvec{\beta })} )$ be the solution to ${{\mathcal {T}}}(y,u,p,p_1) = \varvec{\beta }$. As a consequence of (A5) it is also a solution to $\underline{{{\mathcal {T}}}}(y,u,p,p(0)) = \varvec{\beta }$ with $p \in W_{\infty }$. Thus the first two equations of this latter equality can be expressed as

4.35a $$\begin{aligned} - p_t - {{\mathcal {A}}}^* p - {{\mathcal {F}}}'(\bar{y})^*p + y - [{{\mathcal {F}}}'(\bar{y})^*\bar{p}]'(y - \bar{y})&= \beta _1 \quad \text { in } L^2(I;V^*), \end{aligned}$$

4.35b $$\begin{aligned} \langle \alpha u - B^* p - \beta _2, w - u \rangle _U&\ge 0 \quad \text {for all } w \in U_{ad}, \end{aligned}$$

where we dropped the dependence of $\displaystyle \left( y_{(\varvec{\beta })}, u_{(\varvec{\beta })}, p_{(\varvec{\beta })} \right)$ on $\displaystyle \varvec{\beta }$. Since $p \in \widetilde{W}_{\infty } \subset C({\bar{I}}; Y)$ inequality (4.35b) implies that $u \in C({\bar{I}}; {{\mathcal {U}}})$.

${\underline{\mathrm{Step\,2}: (\mathrm{Boundedness\,of} \{ p_{(\varvec{\beta })}: \varvec{\beta }\in {{\hat{V}}} \}).}}$ Since ${{\hat{V}}}$ is assumed to be bounded, the discussion in Remark 4.2 shows that there exists a constant $M > 0$ such that

$$\begin{aligned} \left\Vert y_{(\varvec{\beta })}\right\Vert _{W_{\infty }} + \left\Vert u_{(\varvec{\beta })}\right\Vert _U \le M \quad \text {for all } \varvec{\beta }\in {{\hat{V}}}. \end{aligned}$$

To argue the boundedness of $p_{(\varvec{\beta })}$, we use similar techniques as in the proof of Proposition 1. In the following ${\tilde{\delta }}, z$ and $C_1$ are taken as in the proof of that proposition.

For arbitrary $r \in R = \left\{ r \in L^2(I;V^*): \left\Vert r\right\Vert _{L^2(I;V^*)} \le {\tilde{\delta }} \right\}$, *z* denotes the solution to

4.36 $$\begin{aligned} z_t - {{\mathcal {A}}}z - {{\mathcal {F}}}'(\bar{y})z - B (w - u) = -r, \ z(0) = 0, \ w = -Kz. \end{aligned}$$

We know that $\left\Vert z\right\Vert _{W_{\infty }} \le C_1$ and $\left\Vert w\right\Vert _{L^{\infty }(I;{{\mathcal {U}}})} \le \eta$ independently of $r \in R$. Due to (4.35a), we have

4.37 $$\begin{aligned} \langle p, r \rangle _{L^2(I;V), L^2(I;V^*)}&= \langle p, -z_t + {{\mathcal {A}}}z + {{\mathcal {F}}}'(\bar{y})z \rangle _{L^2(I;V), L^2(I;V^*)} + \langle B^*p, w - u \rangle _U, \nonumber \\&= \langle y - [{{\mathcal {F}}}'(\bar{y})^*\bar{p}]'(y - \bar{y}) -\beta _1, z \rangle _{L^2(I;V^*), L^2(I;V)}\nonumber \\&\quad + \langle \alpha u - \beta _2, w - u \rangle _{U}, \end{aligned}$$

where we used (4.35b) and the feasibility of $w \in U_{ad}$. Consequently

$$\begin{aligned} \langle p, r \rangle _{L^2(I;V), L^2(I;V^*)}\le & {} \left\Vert z\right\Vert _{L^2(I;V)} \left( \left\Vert y\right\Vert _{L^2(I;V^*)}\right. \\&\left. + \left\Vert \beta _1\right\Vert _{L^2(I;V^*)} + \left\Vert [{{\mathcal {F}}}'(\bar{y})^*\bar{p}]'(y - \bar{y})\right\Vert _{L^2(I;V^*)} \right) \\&+ \left( \alpha \left\Vert u\right\Vert _{U} + \left\Vert \beta _2\right\Vert _U \right) \left\Vert w - u\right\Vert _U. \end{aligned}$$

The right hand side is uniformly bounded for $\varvec{\beta }$ in the bounded set ${{\hat{V}}}$ and w.r.t. $r \in R$. Hence taking the supremum w.r.t. $r \in R$ we verified that $\displaystyle \left\{ \left\Vert p_{(\varvec{\beta })}\right\Vert _{L^2(I;V^*)}: \varvec{\beta }\in {{\hat{V}}} \right\}$ is bounded. Boundedness of $\displaystyle \left\{ \left\Vert p_{(\varvec{\beta })}\right\Vert _{W_{\infty }}: \varvec{\beta }\in {{\hat{V}}} \right\}$ follows from (4.35a).

${\underline{\mathrm{Step\,3}: (\mathrm{Continuity\,of\,p}_{(\varvec{\beta })} \mathrm{in\,W}_{\infty }).}}$ Let $\{ \varvec{\beta }_n \}$ be a convergent sequence in ${{\hat{V}}}$ with limit $\varvec{\beta }$. Since $\displaystyle \left\{ \left\Vert p_{(\varvec{\beta })}\right\Vert _{W_{\infty }}: n \in {\mathbb {N}}\right\}$ is bounded, there exists a subsequence $\{ \varvec{\beta }_{n_k} \}$ such that $\displaystyle p_{(\varvec{\beta }_{n_k})} \rightharpoonup {\tilde{p}}$ weakly in $W_{\infty }$ and strongly $L^2(I;Y)$. Here we need the compactness of $W_{\infty }$ in $L^2(I;Y)$, see e.g. [11, Satz 8.1.12, p. 213]. Passing to the limit in the variational form of

$$\begin{aligned} - \partial _t p_{(\varvec{\beta }_{n_k})} - {{\mathcal {A}}}^* p_{(\varvec{\beta }_{n_k})} - {{\mathcal {F}}}'(\bar{y})^*p_{(\varvec{\beta }_{n_k})} + y - [{{\mathcal {F}}}'(\bar{y})^*\bar{p}]' \left( y_{(\varvec{\beta }_{n_k})} - \bar{y} \right) = (\varvec{\beta }_{n_k})_1, \end{aligned}$$

we obtain

4.38 $$\begin{aligned} - \partial _t {\tilde{p}} - {{\mathcal {A}}}^* {\tilde{p}} - {{\mathcal {F}}}'(\bar{y})^*{\tilde{p}} + y_{(\varvec{\beta })} - [{{\mathcal {F}}}'(\bar{y})^*\bar{p}]'\left( y_{(\varvec{\beta })} - \bar{y} \right) = (\varvec{\beta })_1. \end{aligned}$$

Since the solution to this equation is unique we have $\displaystyle p_{(\varvec{\beta }_n)} \rightharpoonup p_{(\varvec{\beta })}$ weakly in $W_{\infty }$. To obtain strong convergence we set $\delta \varvec{\beta }= \varvec{\beta }_n - \varvec{\beta }, \ \delta p = p_{(\varvec{\beta }_n)} - p_{(\varvec{\beta })}, \ \delta y = y_{(\varvec{\beta }_n)} - y_{(\varvec{\beta })}$. From (4.35a) we derive that

4.39 $$\begin{aligned} - \partial _t (\delta p) - {{\mathcal {A}}}^* (\delta p) - {{\mathcal {F}}}'(\bar{y})^*(\delta p) + (I - [{{\mathcal {F}}}'(\bar{y})^*\bar{p})]'(\delta y) = (\delta \varvec{\beta })_1. \end{aligned}$$

Consider again (4.36) with $r \in R$. Then we obtain

$$\begin{aligned} \langle \delta p, r \rangle _{L^2(I;V), L^2(I;V^*)}= & {} \langle (I - [{{\mathcal {F}}}'(\bar{y})^*\bar{p}]')(\delta y)\\&- (\delta \varvec{\beta })_1, z \rangle _{L^2(I;V^*), L^2(I;V)} + \langle \delta p, BKz - \bar{u} \rangle _{U}. \end{aligned}$$

This implies that for some $C_2 > 0$,

4.40 $$\begin{aligned}&\sup _{r \in R} \ \langle \delta p, r \rangle _{L^2(I;V), L^2(I;V^*)} \nonumber \\&\quad \le C_2 \left( \left\Vert \delta y\right\Vert _{W_{\infty }} + \left\Vert \delta p\right\Vert _{L^2(I;Y)} + \left\Vert (\delta \varvec{\beta })_1\right\Vert _{L^2(I;V^*)} \right) . \end{aligned}$$

Since $\left\Vert \delta y\right\Vert _{W_{\infty }} \rightarrow 0,\ \left\Vert \delta p\right\Vert _{L^2(I;Y)} \rightarrow 0,\ \left\Vert (\delta \varvec{\beta })_1\right\Vert _{L^2(I;V^*)} \rightarrow 0$ for $n \rightarrow 0$ this implies that $\left\Vert \delta p\right\Vert _{L^2(I;V)} \rightarrow 0$. Together with (4.39) this implies that $\displaystyle \lim _{n \rightarrow \infty } \left\Vert \delta p\right\Vert _{W_{\infty }} = 0$. $\square$

#### Proposition 2

Let assumptions (A) hold and let $(\bar{y}, \bar{u})$, and ${\bar{p}}$ denote a local solution and associated adjoint state state to (${\mathcal {P}}$) corresponding to an initial condition ${\bar{y}}_0 \in B_Y({\tilde{\delta }}_2)$. Then there exists $\varepsilon > 0$ and $C > 0$ such that for all $\varvec{{\hat{\beta }}}$ and $\varvec{\beta } \in {\hat{V}} \cap B_{{{\mathcal {Y}}}}(\varepsilon )$

4.41 $$\begin{aligned}&\left\Vert {\hat{y}}_{(\hat{\varvec{\beta }})} - y_{(\varvec{\beta })}\right\Vert _{W_{\infty }} + \left\Vert {\hat{p}}_{(\hat{\varvec{\beta }})} - p_{(\varvec{\beta })}\right\Vert _{W_{\infty }} + \left\Vert u_{(\hat{\varvec{\beta }})} - u_{(\varvec{\beta })}\right\Vert _{C({\bar{I}};{{\mathcal {U}}})}\! \nonumber \\&\quad \le C \left( \left\Vert {\hat{y}}_{(\hat{\varvec{\beta }})} - y_{(\varvec{\beta })}\right\Vert _{W_{\infty }} + \left\Vert u_{(\hat{\varvec{\beta }})} - u_{(\varvec{\beta })}\right\Vert _{U} + \left\Vert \hat{\varvec{\beta }} - \varvec{\beta }\right\Vert _{{{\mathcal {Y}}}} \right) \end{aligned}$$

holds.

#### Proof

Since ${\bar{p}} \in W_{\infty }$ and $\displaystyle \lim _{t \rightarrow \infty } {\bar{p}}(t) = 0$ in *Y*, there exists $T > 0$ such that

$$\begin{aligned} \frac{1}{\alpha }\left\Vert B^*\bar{p}(t)\right\Vert _Y \le \frac{\eta }{2}, \quad \forall t > T. \end{aligned}$$

Since $p(0) = {\bar{p}}$ and since by Lemma 4.8, $\displaystyle \varvec{\beta } \in {{\mathcal {Y}}}\mapsto p_{(\varvec{\beta })} \in W_{\infty } \subset C({\bar{I}};Y)$ is continuous, there exists $\varepsilon > 0$ such that

$$\begin{aligned} \frac{1}{\alpha } \left\Vert B^* p_{(\varvec{\beta })}(t) + \beta _2(t)\right\Vert _{Y} \le \frac{\eta }{4}, \quad \forall t \ge T, \ \forall \varvec{\beta } \in {{\hat{V}}} \cap B_{{{\mathcal {Y}}}}(\varepsilon ). \end{aligned}$$

Consequently the constraints are inactive for these parameter values, i.e. we have

4.42 $$\begin{aligned} u_{(\varvec{\beta })}(t) = \frac{1}{\alpha } \left[ B^* p_{(\varvec{\beta })}(t) + \beta _2(t)\right] , \ \left\Vert u_{(\varvec{\beta })}(t)\right\Vert _{Y} \le \eta , \quad \forall t \ge T, \ \forall \varvec{\beta } \in {{\hat{V}}} \cap B_{{{\mathcal {Y}}}}(\varepsilon ). \end{aligned}$$

We next treat separately the cases [0, *T*) and $[T,\infty )$. We consider first the case $[T,\infty )$ and set $(y,u,p) = \left( y_{(\varvec{\beta })}, u_{(\varvec{\beta })}, p_{(\varvec{\beta })} \right)$, and $\left( {\hat{y}}, {\hat{u}}, {\hat{p}} \right) = \left( {\hat{y}}_{(\varvec{{\hat{\beta }}})}, {\hat{u}}_{(\varvec{{\hat{\beta }}})}, {\hat{p}}_{(\varvec{{\hat{\beta }}})} \right)$. We shall use that

$$\begin{aligned} \left\Vert {\hat{p}} - p\right\Vert _{L^2(T,\infty ; V)} = \sup _{\left\Vert r\right\Vert _{L^2(T,\infty ; V^*)} \le 1} \int _T^{\infty } \langle {\hat{p}}(t) - p(t), r(t) \rangle _{V,V^*}dt. \end{aligned}$$

Let $z \in W(T,\infty )$ be such that,

$$\begin{aligned} z_t - ({{\mathcal {A}}}- BK)z - {{\mathcal {F}}}'(\bar{y})z = r, \ z(T) = 0, \end{aligned}$$

From Lemma 4.6, see also the proof of Proposition 1, we know that there exists a constant $C_1 > 0$ such that $\displaystyle \left\Vert z\right\Vert _{W(T,\infty )} \le C_1 \left\Vert r\right\Vert _{L^2(T,\infty ;V^*)}$. Then we can estimate

$$\begin{aligned}&\left\Vert {\hat{p}} - p\right\Vert _{L^2(T,\infty ; V)}\\&\quad = \sup _{\left\Vert r\right\Vert _{L^2(T,\infty ;V^*)} \le 1} \int _T^{\infty } \langle {\hat{p}} - p, r \rangle _{V,V^*} dt\\&\quad \le \sup _{\left\Vert r\right\Vert \le 1} \int _T^{\infty } - \langle ({\hat{p}}_t - p_t) + {{\mathcal {A}}}^*({\hat{p}} - p) + {{\mathcal {F}}}'(\bar{y})^*({\hat{p}} - p), z \rangle _{V^*,V} dt \nonumber \\&\qquad + \sup _{\left\Vert r\right\Vert \le 1} \int _T^{\infty } \langle B^*({\hat{p}} - p), Kz \rangle _{V,V^*} dt. \end{aligned}$$

In the following, $C_i$ denote constants independent of $\varvec{{\hat{\beta }}} \text { and } \varvec{\beta } \in {\hat{V}} \cap B_{{{\mathcal {Y}}}}(\varepsilon )$. From (4.35a) and (4.42) we obtain, for $C_2 > 0$,

$$\begin{aligned}&\left\Vert {\hat{p}} - p\right\Vert _{L^2(T,\infty ; V)} \\&\quad \le C_2 \sup _{\left\Vert r\right\Vert \le 1} \int _T^{\infty } \left[ \left\Vert {\hat{y}} - y\right\Vert _{V^*} + \left\Vert [{{\mathcal {F}}}'(\bar{y})^*\bar{p}]'({\hat{y}} - y)\right\Vert _{V^*} + \left\Vert {\hat{\beta }}_1 - \beta _1\right\Vert _{V^*} \right. \\&\qquad \left. + \left\Vert {\hat{\beta }}_2 - \beta _2\right\Vert _{U \cap C(I;{{\mathcal {U}}})} + \alpha \left\Vert B^*\right\Vert \left\Vert K\right\Vert \left\Vert {\hat{u}} - u\right\Vert \right] \left\Vert z\right\Vert _V dt. \end{aligned}$$

From (A3) recall that $\displaystyle \left\Vert [{{\mathcal {F}}}'(\bar{y})^*\bar{p}]'({\hat{y}} - y)\right\Vert _{L^2(I;V^*)} \le C_3 \left\Vert {\hat{y}} - y\right\Vert _{W_{\infty }}$. This gives the following estimate for $C_4 > 0$,

4.43 $$\begin{aligned} \left\Vert {\hat{p}} - p\right\Vert _{L^2(T,\infty ; V)} \le C_4 \left( \left\Vert {\hat{y}} - y\right\Vert _{W_{\infty }} + \left\Vert {\hat{u}} - u\right\Vert _{U} + \left\Vert \hat{\varvec{\beta }} - \varvec{\beta }\right\Vert _{{{\mathcal {Y}}}} \right) . \end{aligned}$$

By (4.35a) we have $\displaystyle \left( {\hat{p}}_t - p_t \right) \in L^2(T, \infty ;V^*)$. Then we obtain ${\hat{p}} - p \in W(T,\infty )$. Then there exists $C_5 > 0$ independent of $\varvec{{\hat{\beta }}}\text { and } \varvec{\beta } \in {\hat{V}} \cap B_{{{\mathcal {Y}}}}(\varepsilon )$ such that,

4.44 $$\begin{aligned} \left\Vert {\hat{p}} - p\right\Vert _{W(T,\infty )} \le C_5 \left( \left\Vert {\hat{y}} - y\right\Vert _{W_{\infty }} + \left\Vert {\hat{u}} - u\right\Vert _{U} + \left\Vert \hat{\varvec{\beta }} - \varvec{\beta }\right\Vert _{{{\mathcal {Y}}}} \right) . \end{aligned}$$

By the embedding $\displaystyle W(T,\infty ) \subset C(T,\infty ;Y)$, there exists a constant $C_6 > 0$:

4.45 $$\begin{aligned} \left\Vert {\hat{p}} - p\right\Vert _{C([T,\infty );Y)} \le C_6 \left( \left\Vert {\hat{y}} - y\right\Vert _{W_{\infty }} + \left\Vert {\hat{u}} - u\right\Vert _{U} + \left\Vert \hat{\varvec{\beta }} - \varvec{\beta }\right\Vert _{{{\mathcal {Y}}}} \right) . \end{aligned}$$

Similarly, we estimate on [0, *T*]:

4.46 $$\begin{aligned} \left\Vert {\hat{p}} - p\right\Vert _{L^2(0,T; V)} = \sup _{\left\Vert r\right\Vert _{L^2(0,T; V^*)} \le 1} \int _0^{T} \langle {\hat{p}} - p, r \rangle _{V,V^*} dt. \end{aligned}$$

Choose *z* as

$$\begin{aligned} z_t - \left( {{\mathcal {A}}}z + {{\mathcal {F}}}'(\bar{y})z \right) = r, \ z(0) = 0, \end{aligned}$$

Then there exists $\displaystyle C_7 > 0$ such that $\displaystyle \left\Vert z\right\Vert _{W(0,T)} \le C_7 \left\Vert r\right\Vert _{L^2(0,T; V^*)}$ by Lemma 4.6. Note that $C_7$ depends on *T*, but *T* is fixed. We obtain the following estimate,

$$\begin{aligned}&\left\Vert {\hat{p}} - p\right\Vert _{L^2(0,T; V)} \\&\quad \le \sup _{\left\Vert r\right\Vert \le 1} \int _0^T - \langle ({\hat{p}}_t - p_t) + {{\mathcal {A}}}^*({\hat{p}} - p) + {{\mathcal {F}}}'(\bar{y})^*({\hat{p}} - p), z \rangle _{V^*,V} dt \\&\qquad + \left\Vert {\hat{p}}(T) - p(T)\right\Vert _Y\left\Vert z(T)\right\Vert _Y. \end{aligned}$$

Then by a similar computation to that for the $t \in [T, \infty )$ case, we obtain,

4.47 $$\begin{aligned} \left\Vert {\hat{p}} - p\right\Vert _{L^2(0,T; V)}\le & {} C_8 \left( \left\Vert {\hat{y}} - y\right\Vert _{W_{\infty }} + \left\Vert {\hat{\beta }}_1 - \beta _1\right\Vert _{L^2(I;V^*)} \right) \nonumber \\&+ \left\Vert {\hat{p}}(T) - p(T)\right\Vert _Y\left\Vert z(T)\right\Vert _Y. \end{aligned}$$

By (4.45) with $\left\Vert z(T)\right\Vert _Y \le C_9$, we obtain

$$\begin{aligned} \left\Vert {\hat{p}}(T) - p(T)\right\Vert _Y\left\Vert z(T)\right\Vert _Y \le C_7 C_9 \left( \left\Vert {\hat{y}} - y\right\Vert _{W_{\infty }} + \left\Vert {\hat{u}} - u\right\Vert _{U} + \left\Vert \hat{\varvec{\beta }} - \varvec{\beta }\right\Vert _{{{\mathcal {Y}}}} \right) . \end{aligned}$$

Combining this estimate with (4.43) and (4.47), we obtain for some $C_{10} > 0$

4.48 $$\begin{aligned} \left\Vert {\hat{p}}_{(\hat{\varvec{\beta }})} - p_{(\varvec{\beta })}\right\Vert _{W_{\infty }} \le C_{10} \left( \left\Vert {\hat{y}}_{(\hat{\varvec{\beta }})} - y_{(\varvec{\beta })}\right\Vert _{W_{\infty }} + \left\Vert {\hat{u}}_{(\hat{\varvec{\beta }})} - u_{(\varvec{\beta })}\right\Vert _{U} + \left\Vert \hat{\varvec{\beta }} - \varvec{\beta }\right\Vert _{{{\mathcal {Y}}}} \right) . \end{aligned}$$

We also have

$$\begin{aligned} u_{(\varvec{\beta })} = {\mathbb {P}}_{{\mathcal {U}}_{ad}} \left[ -\frac{1}{\alpha }\left( B^* p_{(\varvec{\beta })} + \beta _2 \right) \right] \in U \cap C({\bar{I}};{{\mathcal {U}}}), \end{aligned}$$

and thus

$$\begin{aligned}&\left\Vert {{\hat{u}}}_{(\hat{\varvec{\beta }})}(t) - u_{(\varvec{\beta })}(t)\right\Vert _{{{\mathcal {U}}}} \\&\quad \le \left\Vert {\mathbb {P}}_{{\mathcal {U}}_{ad}} \left[ -\frac{1}{\alpha } \left( B^*{\hat{p}}_{(\hat{\varvec{\beta }})}(t) + {\hat{\beta }}_2(t) \right) \right] - {\mathbb {P}}_{{\mathcal {U}}_{ad}} \left[ -\frac{1}{\alpha }\left( B^* p(t)_{(\varvec{\beta })} + \beta _2(t) \right) \right] \right\Vert _{{{\mathcal {U}}}}\\&\quad \le \frac{1}{\alpha } \left( \left\Vert B^*\right\Vert \left\Vert {\hat{p}}_{(\hat{\varvec{\beta }})}(t) - p(t)_{(\varvec{\beta })}\right\Vert _{Y} + \left\Vert {\hat{\beta }}_2(t) - \beta _2(t)\right\Vert _{{{\mathcal {U}}}} \right) . \end{aligned}$$

This yields

4.49 $$\begin{aligned} \left\Vert {{\hat{u}}}_{(\hat{\varvec{\beta }})} - u_{(\varvec{\beta })}\right\Vert _{C(\bar{I};{{\mathcal {U}}})} \le C_{11} \left( \left\Vert {\hat{p}}_{(\hat{\varvec{\beta }})} - p_{(\varvec{\beta })}\right\Vert _{W_{\infty }} + \left\Vert {\hat{\beta }}_2 - \beta _2\right\Vert _{C({\bar{I}};{{\mathcal {U}}})} \right) , \end{aligned}$$

and (4.41) follows. Combining Remark 4.2 and (4.41) there exists a constant *L* such that

4.50 $$\begin{aligned} \left\Vert {\hat{y}}_{(\hat{\varvec{\beta }})} - y_{(\varvec{\beta })}\right\Vert _{W_{\infty }} + \left\Vert {\hat{p}}_{(\hat{\varvec{\beta }})} - p_{(\varvec{\beta })}\right\Vert _{W_{\infty }} + \left\Vert {\hat{u}}_{(\hat{\varvec{\beta }})} - u_{(\varvec{\beta })}\right\Vert _{U \cap C(\bar{I};Y)} \le L \left\Vert \hat{\varvec{\beta }} - \varvec{\beta }\right\Vert _{{{\mathcal {Y}}}}\nonumber \\ \end{aligned}$$

for all $\displaystyle \varvec{{\hat{\beta }}}$ and $\displaystyle \varvec{\beta } \in {\hat{V}} \cap B_{{{\mathcal {Y}}}}(\varepsilon )$. Here and in the following the $p_1$ coordinate of the adjoint state coincides with *p*(0). Therefore it is not indicated. $\square$

With the above proposition, the verification of (H1)–(H7) is concluded. We also obtain the following corollary to Theorem 2.1.

#### Corollary 4.9

Let assumptions (A) hold and let $(\bar{y}, \bar{u})$ be a local solution of (${\mathcal {P}}$) corresponding to an initial datum ${\bar{y}}_0 \in B_Y(\tilde{\delta }_2)$. Then there exist $\delta _3 > 0$, a neighborhood ${{\hat{U}}} = {{\hat{U}}}(\bar{y}, \bar{u}, p) \subset W_{\infty } \times (U \cap C(\bar{I};{{\mathcal {U}}})) \times W_{\infty }$, and a constant $\mu > 0$ such that for each $y_0 \in B_Y({\bar{y}}_0, \delta _3)$ there exists a unique $(y(y_0), u(y_0), p(y_0)) \in {{\hat{U}}}$ satisfying the first order condition, and

4.51 $$\begin{aligned}&\left\Vert \left( y({\hat{y}}_0), u({\hat{y}}_0)), p({\hat{y}}_0)) \right) - \left( y({\tilde{y}}_0), u({\tilde{y}}_0), p({\tilde{y}}_0)) \right) \right\Vert _{W_{\infty } \times (U \cap C(\bar{I};{{\mathcal {U}}})) \times W_{\infty }} \nonumber \\&\quad \le \mu \left\Vert {\hat{y}}_0 - {\tilde{y}}_0\right\Vert _{Y}, \end{aligned}$$

for all ${\hat{y}}_0, {\tilde{y}}_0 \in B_Y({\bar{y}}_0,\delta _3)$, and $\left( y(y_0), u(y_0) \right)$ is a local solution of (${\mathcal {P}}$).

Next we obtain one of the main results of this paper, the Fréchet differentiability of the local value function associated to (${\mathcal {P}}$). By referring to a local value function we pay attention to the fact that for some $y_0\in B_Y({\tilde{\delta }}_2)$, problem (${\mathcal {P}}$) may not admit a unique solution. But since due to the second order optimality condition local solutions are locally unique under small perturbations of $y_0$, there is a well-defined local value function. We continue to use the notation for ${{\hat{U}}}$ and $B_Y({\bar{y}}_0, \delta _3)$ of Corollary 4.9.

#### Theorem 4.10

(Sensitivity of Cost) Let assumptions (A) hold and let $(\bar{y}, \bar{u})$ be a local solution of (${\mathcal {P}}$) corresponding to an initial datum $\bar{y}_0 \in B_Y({\tilde{\delta }}_2)$. Then for each $y_0 \in B_Y({\bar{y}}_0, \delta _3)$ the local value function ${\mathcal {V}}$ associated to (${\mathcal {P}}$) is Fréchet differentiable with derivative given by

4.52 $$\begin{aligned} {\mathcal {V}}'(y_0) = - p(0;y_0). \end{aligned}$$

#### Proof

Let ${\bar{y}}_0 \in B_Y({\tilde{\delta }}_2), y_0 \in B_Y({\bar{y}}_0, \delta _3)$, and choose $\delta y_0$ sufficiently small so that $y_0 + \delta y_0 \in B_Y({\bar{y}}_0, \delta _3)$ as well. Following Corollary 4.9 let $({\tilde{y}}(y_0 + s(\delta y_0)), {\tilde{u}}(y_0 + s(\delta y_0)), {\tilde{p}}(y_0 + s(\delta y_0)))\in {{\hat{U}}}$ for $s\in [0,1]$ be solutions of the optimality system with $({\tilde{y}}(y_0 + s(\delta y_0)), {\tilde{u}}(y_0 + s(\delta y_0)))$ local solutions to (${\mathcal {P}}$). We obtain

4.53 $$\begin{aligned} {\mathcal {V}}(y_0 + s(\delta y_0)) - {\mathcal {V}}(y_0)= & {} \left( \frac{1}{2} \left\Vert {\tilde{y}}\right\Vert ^2_{L^2(I,Y)} + \frac{\alpha }{2} \left\Vert {\tilde{u}}\right\Vert ^2_U\right) - \left( \frac{1}{2} \left\Vert y\right\Vert ^2_{L^2(I,Y)} + \frac{\alpha }{2} \left\Vert u\right\Vert ^2_U\right) \nonumber \\= & {} \langle y, {\tilde{y}} - y \rangle _{L^2(I,Y)} + \alpha \langle u, {\tilde{u}} - u \rangle _U\nonumber \\&+ \frac{1}{2} \left\Vert {\tilde{y}} - y\right\Vert ^2_{L^2(I,Y)} + \frac{{\alpha }}{2} \left\Vert {\tilde{u}} - u\right\Vert ^2_{U}. \end{aligned}$$

Observe the identity

$$\begin{aligned}&\langle y, {\tilde{y}} - y \rangle _{L^2(I,Y)} + \alpha \langle u, {\tilde{u}} - u \rangle _U\\&\quad = {-}( p(0), s(\delta y_0) )_Y {-} \langle ({\tilde{y}}_t - y_t) - {{\mathcal {A}}}( {\tilde{y}} - y) - {{\mathcal {F}}}'(y)({\tilde{y}} - y), p \rangle \\&\qquad + \alpha \langle u, {\tilde{u}} - u \rangle _U\\&\quad = {-}( p(0), s(\delta y_0) )_Y {-} \langle {{\mathcal {F}}}({\tilde{y}}) - {{\mathcal {F}}}(y) - {{\mathcal {F}}}'(y)({\tilde{y}} - y), p \rangle _{L^2(I;V^*), L^2(I;V)}\\&\qquad + \langle \alpha u - B^*p, {\tilde{u}} - u \rangle _U, \end{aligned}$$

where $p=p(y_0)$. Now we have for $\displaystyle {\mathcal {V}}(y_0 + s(\delta y_0)) - {\mathcal {V}}(y_0)$,

4.54 $$\begin{aligned}&{\mathcal {V}}(y_0 + {s}(\delta y_0)) - {\mathcal {V}}(y_0)\nonumber \\&\quad = {-}( p(0), {s}(\delta y_0) )_Y + \langle {{\mathcal {F}}}(y) - {{\mathcal {F}}}({\tilde{y}}) + {{\mathcal {F}}}'(y)({\tilde{y}} - y), p \rangle _{L^2(I,V^*),L^2(I,V)} \nonumber \\&\qquad + \langle \alpha u - B^*p, {\tilde{u}} - u \rangle _{U} + \frac{1}{2} \left\Vert {\tilde{y}} - y\right\Vert ^2_{L^2(I,Y)} + \frac{{\alpha }}{2} \left\Vert {\tilde{u}} - u\right\Vert ^2_{U}. \end{aligned}$$

Since $p \in L^2(I;V)$, $\left\Vert {\tilde{y}} - y\right\Vert _{W_{\infty }} = O(s)$, and by the continuous Fréchet differentiability of ${{\mathcal {F}}}'$ due to (A3) we have

4.55 $$\begin{aligned} \left|\langle {{\mathcal {F}}}({\tilde{y}}) - {{\mathcal {F}}}(y) + {{\mathcal {F}}}'(y)({\tilde{y}} - y), p \rangle _{L^2(I,V^*),L^2(I,V)}\right| = o(s) \end{aligned}$$

Let $s_n \rightarrow 0$ be an arbitrary convergent sequence. By Corollary 4.9 we have that

$$\begin{aligned} \left\Vert {\tilde{u}}(y_0 + s_n(\delta y_0)) - u(y_0)\right\Vert _U \le \mu s_n(\delta y_0) \end{aligned}$$

for all $s_n$ sufficiently small. Hence there exists a subsequence, denoted by the same notation and some $\dot{u}$ such that

$$\begin{aligned} s_n^{-1}\left( {\tilde{u}}(y_0 + s_n(\delta y_0)) - u(y_0)\right) \rightharpoonup \dot{u} \text { weakly in } U. \end{aligned}$$

Using (4.18), we have

$$\begin{aligned} \lim _{n \rightarrow \infty } s_n^{-1} \langle \alpha u - B^*p, {{\tilde{u}}} - u \rangle _U = \langle \alpha u - B^*p, \dot{u} \rangle _U \ge 0. \end{aligned}$$

Analogously

$$\begin{aligned} \lim _{n \rightarrow \infty } s_n^{-1} \langle \alpha {{\tilde{u}}} - B^*p, u - {{\tilde{u}}} \rangle _U = \langle \alpha u - B^*p, \dot{u} \rangle _U \le 0. \end{aligned}$$

and hence $\langle \alpha u - B^*p, \dot{u} \rangle _U = 0$. Since the sequence $\{ s_n \}$ is arbitrary, we obtain

4.56 $$\begin{aligned} \langle \alpha u - B^*p, {{\tilde{u}}} - u \rangle _U = o(s). \end{aligned}$$

Corollary 4.9 yields,

4.57 $$\begin{aligned} \left\Vert {\tilde{y}}(y_0 + {s_n}(\delta y_0)) - y(y_0)\right\Vert ^2_{L^2(I;Y)} + \alpha \left\Vert {\tilde{u}}(y_0 + {s_n}(\delta y_0)) - u(y_0)\right\Vert ^2_{L^2(I;Y)} = o(s_n). \end{aligned}$$

Combining (4.55), (4.56), and (4.57) we obtain

4.58 $$\begin{aligned} \lim _{s \rightarrow 0^+} {s^{-1}}\left( {\mathcal {V}}(y_0 + s(\delta y_0)) - {\mathcal {V}}(y_0)\right) = {-}( p(0), (\delta y_0) )_Y. \end{aligned}$$

This implies the Gateaux differentiability. Since $y_0 \rightarrow p(y_0)$ is continuous from $B_Y({\bar{y}}_0;\delta _3)$ to $C({\bar{I}},Y)$ the mapping $y_0\rightarrow {\mathcal {V}}(y_0)$ is Fréchet differentiable in $B_Y({\bar{y}}_0;\delta _3)$.

$$\square $$

#### Remark 4.4

(Sensitivity w.r.t. other parameters) We have developed a technique to verify the continuous differentiability of the local value function ${\mathcal {V}}$ pertaining to a semilinear parabolic equation on infinite time horizon subject to control constraints with respect to small initial data $y_0 \in Y$. Thus the parameter *q* in ($P_q$) is the initial condition $y_0$. The reason to focus on this case is due to feedback control. Without much additional effort the sensitivity analysis of the value function could be carried out with respect to other parameters as for instance additive noise on the right hand side of the state equation. The papers cited in the introduction, see e.g. [14, 15], consider such situations for the finite horizon case.

## Proof of Theorem 3.2: Derivation of the HJB Equation

Utilizing the results established so far we now verify that the (global) value function ${\mathcal {V}}$ (i.e. the value function associated to global minima) is a solution to a Hamilton–Jacobi–Bellman equation. The initial conditions will be chosen from the neighborhood $Y_0$ of the origin in *Y* so that the assertions of Theorem 4.10 and Corollary 4.9 are available. It will be convenient to recall the dynamic programming principle for the infinite time horizon problem: let $y_0$ be an initial condition for which a solution to (${\mathcal {P}}$) exists. Then for all $\tau > 0$, we have

5.1 $$\begin{aligned} {\mathcal {V}}(y_0) = \inf _{u \in L^2(0,\tau ;{\mathcal {U}}_{ad})} \int _{0}^{\tau } \ell (S(u,y_0;t),u(t)) dt + {\mathcal {V}}(S(u,y_0;\tau )), \end{aligned}$$

where $\displaystyle \ell (y,u) = \frac{1}{2} \left\Vert y\right\Vert ^2_Y + \frac{\alpha }{2} \left\Vert u\right\Vert ^2_{{{\mathcal {U}}}}$, and $S(u,y_0;t)$ denotes the solution to (3.2b), (3.2c) on $(0,\tau ]$.

For convenience we restate Theorem 3.2. Utilizing the notation that we have already established we can now slightly ease the assumption on the regularity of ${{\mathcal {F}}}({\bar{y}})$.

### Theorem 5.1

Let assumptions (A) hold and let $(\bar{y}, \bar{u})$ be a global solution of (${\mathcal {P}}$) corresponding to an initial datum ${\bar{y}}_0 \in B_Y(\tilde{\delta }_2)$. Let $Y_0$ denote the subset of initial conditions in $B_Y({\bar{y}}_0, \delta _3)$ which allow global solutions in ${{\hat{U}}}$, and assume that for each $y_0\in {{\mathcal {D}}}({{\mathcal {A}}}) \cap Y_0$ there exists $T_{y_0}>0$ such that ${{\mathcal {F}}}({\bar{y}})\in C([0,T_{y_0});Y)$. Then the following Hamilton–Jacobi–Bellman equation holds at $y_0$:

5.2 $$\begin{aligned}&{\mathcal {V}}'(y)({{\mathcal {A}}}y + {{\mathcal {F}}}(y)) + \frac{1}{2} \left\Vert y\right\Vert ^2_Y + \frac{\alpha }{2} \left\Vert {\mathbb {P}}_{{\mathcal {U}}_{ad}} \left( -\frac{1}{\alpha }B^*{\mathcal {V}}'(y) \right) \right\Vert ^2_Y\nonumber \\&\quad + \left\langle B^* {\mathcal {V}}'(y),{\mathbb {P}}_{{\mathcal {U}}_{ad}} \left( -\frac{1}{\alpha }B^*{\mathcal {V}}'(y) \right) \right\rangle _Y = 0. \end{aligned}$$

If for the optimal trajectory ${\bar{y}}(t) \in B_Y({\bar{y}}_0, \delta _3)\cap {{\mathcal {D}}}({{\mathcal {A}}})$ for a.a. $t\in (0,\infty$) and $T_{y_0}=\infty$, then (5.2) holds at a.a. $t\in (0,\infty$) and

5.3 $$\begin{aligned} {{\bar{u}}}(t) = {\mathbb {P}}_{{\mathcal {U}}_{ad}} \left( -\frac{1}{\alpha } B^*{\mathcal {V}}'({{\bar{y}}}(t)) \right) . \end{aligned}$$

### Proof

The proof is similar to that of [5, Proposition 10]. For the sake of completeness and since it also requires some changes we provide it here. Choose and fix some $y_0 \in {{\mathcal {D}}}({{\mathcal {A}}}) \cap Y_0$. Then the existence of a (globally) optimal pair $({\hat{y}}, {\hat{u}}) \in W_{\infty } \times U_{ad}$ to (${\mathcal {P}}$) and of an associated adjoint state ${{\hat{p}}}\in W_\infty$ with $({{\hat{y}}},{{\hat{u}}}, {{\hat{p}}}) \in {{\hat{U}}}$ are guaranteed, see Corollary 4.9. In particular we have that $\displaystyle {\hat{u}}(t) = {\mathbb {P}}_{{\mathcal {U}}_{ad}} \left( \frac{1}{\alpha }B^*{{{\hat{p}}}}(t) \right)$, and since $\displaystyle {{\hat{p}}} \in C([0,\infty );Y)$ we have that $\displaystyle {{\hat{u}}}\in C([0,\infty );Y)$. Let $u_0$ denote the limit of ${\hat{u}}$ as time *t* tends to 0. Since ${\hat{y}} \in C([0,\infty ); Y)$ and since $B_Y(y_0,\delta _3)$ is open there exists $\tau _{y_0} > 0$ such that ${\hat{y}}(t) \in B_Y(y_0,\delta _3)$, for all $t \in [0,\tau _{y_0})$.

${\underline{\mathrm{Step\,1}:}}$ Let us first prove that

5.4 $$\begin{aligned} {\mathcal {V}}'(y_0) \big ({{\mathcal {A}}}y_0 + {{\mathcal {F}}}(y_0) + B u_0 \big ) + \ell (y_0, u_0) = 0. \end{aligned}$$

For this purpose we invoke the dynamic programing principle: We have

5.5 $$\begin{aligned} \frac{1}{\tau } \int _{0}^{\tau } \ell ({\hat{y}}(s), {\hat{u}}(s))ds + \frac{1}{\tau } \big ( {\mathcal {V}}({\hat{y}}(\tau )) - {\mathcal {V}}(y_0) \big ) = 0, \end{aligned}$$

where we choose $\tau \in (0,\min (T_{y_0}, \tau _{y_0}))$ . By continuity of ${\hat{y}}$ and ${\hat{u}}$ at time 0, the first term converges to $\displaystyle \ell (y_0, u_0)$ as $\tau \rightarrow 0$. To take $\tau \rightarrow 0$ in the second term we first consider

5.6 $$\begin{aligned} \frac{1}{\tau }\big ( {\hat{y}}(\tau ) - y_0 \big ) = \frac{1}{\tau }\big (e^{{{\mathcal {A}}}\tau }y_0 - y_0 \big ) + \frac{1}{\tau } \int _{0}^{\tau } e^{{{\mathcal {A}}}(\tau - s)} \big [ {{\mathcal {F}}}({\hat{y}}(s)) + B {\hat{u}}(s) \big ] ds. \end{aligned}$$

Using the facts that $y_0\in {{\mathcal {D}}}({{\mathcal {A}}})$, that the terms in square brackets are continuous with values in *Y*, and that ${{\mathcal {A}}}$ generates a strongly continuous semigroup on *Y*, we can pass to the limit in (5.6) to obtain that

5.7 $$\begin{aligned} \lim _{\tau \rightarrow 0^+}\frac{1}{\tau }\big ( {\hat{y}}(\tau ) - y_0 \big ) = {{\mathcal {A}}}y_0 + {{\mathcal {F}}}(y_0) + B u_0 \text { in } Y. \end{aligned}$$

Now we return to the second term in (5.5) which we express as

5.8 $$\begin{aligned} \frac{1}{\tau } \big ( {\mathcal {V}}({\hat{y}}(\tau )) - {\mathcal {V}}(y_0) \big )= \int _{0}^{1} {\mathcal {V}}' \big (y_0 + s ( {\hat{y}}(\tau ) - y_0 ) \big )ds \; \frac{1}{\tau }( {\hat{y}}(\tau ) - y_0 ). \end{aligned}$$

Using (5.7) and since $y\rightarrow {\mathcal {V}}' (y)$ is continuously differentiable at $y_0$, we can pass to the limit in (5.8) to obtain

5.9 $$\begin{aligned} \lim _{\tau \rightarrow 0^+}\frac{1}{\tau } \big ( {\mathcal {V}}({\hat{y}}(\tau )) - {\mathcal {V}}(y_0) \big )= {\mathcal {V}}'(y_0) \big ({{\mathcal {A}}}y_0 + {{\mathcal {F}}}(y_0) + B u_0 \big ). \end{aligned}$$

Now we can pass to the limit in (5.5) and obtain (5.4).

${\underline{\mathrm{Step\,2}:}}$ For $u \in {\mathcal {U}}_{ad}$ we define ${\tilde{u}}\in U_{ad}$ by,

$$\begin{aligned} \widetilde{u}(\tau ,x) = {\left\{ \begin{array}{ll} u \quad \text {for } \tau \in (0,1) \\ 0 \quad \text { for } \tau \in [1, \infty ) \end{array}\right. } \end{aligned}$$

and define ${\tilde{y}} = S(y_0,{\tilde{u}})$ as the solution to (3.2b), (3.2c). Then ${{\tilde{y}}}(t) \in B_Y(\bar{y}_0,\delta _3)$, for all *t* sufficiently small, and by (5.1) we have,

$$\begin{aligned} \frac{1}{\tau } \int _{0}^{\tau } \ell ({\tilde{y}}(s), u(s))ds + \frac{1}{\tau } \big ( {\mathcal {V}}({\tilde{y}}(\tau )) - {\mathcal {V}}(y_0) \big ) \ge 0, \end{aligned}$$

for all $\tau$ sufficiently small. We pass to the limit $\tau \rightarrow 0^+$ with the same arguments as in Step 1 and obtain

5.10 $$\begin{aligned} {\mathcal {V}}'(y_0) \big ({{\mathcal {A}}}y_0 + {{\mathcal {F}}}(y_0) + B u \big ) + \ell (y_0, u) \ge 0. \end{aligned}$$

This inequality becomes an equality if $u = u_0$, and thus the quadratic function on the left had side of (5.10) reaches its minimum 0 at $u = u_0$. This implies that

$$\begin{aligned} u_0 = {\mathbb {P}}_{{\mathcal {U}}_{ad}} \left( -\frac{1}{\alpha } B^* {\mathcal {V}}'(y_0) \right) . \end{aligned}$$

Inserting this expression into (5.4) we obtain

5.11 $$\begin{aligned}&{\mathcal {V}}'(y_0)({{\mathcal {A}}}y_0 + {{\mathcal {F}}}(y_0)) + \frac{1}{2} \left\Vert y_0\right\Vert ^2_Y + \frac{\alpha }{2}\left\Vert {\mathbb {P}}_{{\mathcal {U}}_{ad}}\left( -\frac{1}{\alpha } B^* {\mathcal {V}}'(y_0) \right) \right\Vert ^2_Y\nonumber \\&\quad + \left\langle B^* {\mathcal {V}}'(y_0),{\mathbb {P}}_{{\mathcal {U}}_{ad}}\left( -\frac{1}{\alpha } B^* {\mathcal {V}}'(y_0) \right) \right\rangle _Y = 0. \end{aligned}$$

Under the additional assumptions on the trajectory, (5.3) follows.

$$\square $$

## Some Applications

In this section we discuss the applicability of the framework in two specific cases. It should be noted that even for linear state equations, the sensitivity result for the constraint infinite horizon optimal control problem may be new.

### Fisher’s Equation

We consider the optimal stabilization problem for the Fisher equation in an open connected bounded domain $\Omega$ in ${\mathbb {R}}^d, \ d \in \{1,2,3,4\}$, with Lipschitzian boundary $\Gamma = \partial \Omega$:

6.1a $$\begin{aligned} ({\mathcal {P}}_{Fis}) \qquad {\mathcal {V}}(y_0) = \min _{\begin{matrix} (y,u) \in W_\infty \times U_{ad} \end{matrix}} \ \frac{1}{2} \int _{0}^{\infty } \left\Vert y\right\Vert ^2_Y dt + \frac{\alpha }{2} \int _{0}^{\infty } \left\Vert u\right\Vert ^2_{{{\mathcal {U}}}} dt, \end{aligned}$$

subject to 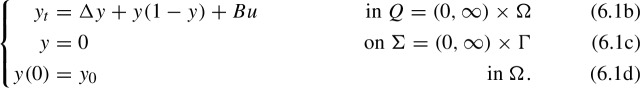 where ${{\mathcal {U}}}$ and $U_{ad}$ are as in Sect. 3.1, $B \in {{\mathcal {L}}}({{{\mathcal {U}}}, Y})$, with $Y=L^2(\Omega )$ and $V = H^1_0(\Omega )$. To further cast this problem in the framework of Sect. 3, we define the operator

$$\begin{aligned} {{\mathcal {A}}}y = (\Delta + \mathbf {I}) y \quad \text {and} \quad y|_{\Gamma } = 0, \quad {{\mathcal {D}}}({{\mathcal {A}}}) = H^2(\Omega ) \cap V. \end{aligned}$$

Clearly ${{\mathcal {A}}}$ has an extension as operator ${{\mathcal {A}}}\in {{\mathcal {L}}}(V,V^*)$. Moreover it generates an analytic semigroup on *Y*. Thus (A1) holds. For ${{\mathcal {U}}}= Y$ and $B = \mathbf {I}$, condition (A2) is trivially satisfied. Feedback stabilization by finite dimensional controllers was analyzed in [24], for example.

It can readily be checked that the nonlinearity ${{\mathcal {F}}}(y) = -y^2$ is twice continuously differentiable as mapping ${{\mathcal {F}}}: W_{\infty } \rightarrow L^2(I;V^*)$. The first and second derivatives of ${{\mathcal {F}}}$ are given by,

$$\begin{aligned} {{\mathcal {F}}}'(y)v_1 = 2yv_1, \quad {{\mathcal {F}}}''(y)(v_1,v_2) = 2(v_1,v_2), \quad \text { for } y,v_1,v_2 \in W_{\infty }. \end{aligned}$$

Since the second derivative is independent of *y*, its boundedness is automatic. For the sake of illustration we verify the boundedness of the bilinear form of the second derivative on $W_\infty \times W_\infty$. For this purpose, for arbitrary $y\in W_\infty , v_1, v_2\in W_\infty , \phi \in L^2(I;V)$ we estimate

6.2 $$\begin{aligned} \begin{aligned}&\int _{0}^{\infty } \langle {{\mathcal {F}}}''({y})(v_1, v_2), \phi \rangle _{V^*,V} dt\\&\quad \le 2 \int _{0}^{\infty } \int _{\Omega } v_1 v_2 \phi \ dx dt \le \int ^\infty _0 \left\Vert v_1\right\Vert _{L^2(\Omega )} \left\Vert v_2\right\Vert _{L^4(\Omega )} \left\Vert \phi \right\Vert _{L^4(\Omega )}\,dt,\\&\quad \le C_1 \left\Vert v_1\right\Vert _{W_{\infty }} \int ^\infty _0 \left\Vert v_2\right\Vert _{V} \left\Vert \phi \right\Vert _{V}\,dt \le C_2 \left\Vert v_1\right\Vert _{W_{\infty }} \left\Vert v_2\right\Vert _{L^2(I;V)} \left\Vert \phi \right\Vert _{L^2(I;V)},\\&\quad \le C_3 \left\Vert v_1\right\Vert _{W_{\infty }} \left\Vert v_2\right\Vert _{W_{\infty }} \left\Vert \phi \right\Vert _{L^2(I;V)}, \end{aligned}\nonumber \\ \end{aligned}$$

where $C_i$ are embedding constants, independent of $y\in W_\infty , v\in W_\infty , \phi \in L^2(I;V)$. We use that *V* embeds continuously into $L^4(\Omega )$ in dimension up to 4. This implies that $\left\Vert {{\mathcal {F}}}''({y})(v_1, v_2)\right\Vert _{L^2(I;V^*)} \le C_3 \left\Vert v_1\right\Vert _{W_{\infty }} \left\Vert v_2\right\Vert _{W_{\infty }}$. Finally we have ${{\mathcal {F}}}(0) = {{\mathcal {F}}}'(0) = 0$ and thus (A3) and (3.3) are satisfied.

Turning to (A4) we show that ${{\mathcal {F}}}(y):W(0,T) \rightarrow L^1(0,T;V^*)$ is continuous for every $T > 0$. We consider the sequence $\displaystyle y_n \rightharpoonup {\hat{y}}$ in $W_{\infty }$ and let $z \in L^{\infty }(0,T; V)$ be given. Then we estimate

$$\begin{aligned}&\int _{0}^T \langle {{\mathcal {F}}}(y_n) - {{\mathcal {F}}}({\hat{y}}), z \rangle _{V^*,V} dt\\&\quad = \int _{0}^T \langle y_n^2 - {\hat{y}}^2, z \rangle _{V^*,V} = \int _{0}^T \int _{\Omega }(y_n - {\hat{y}})(y_n + {\hat{y}})z \ dx dt\\&\quad \le C_4 \int _{0}^T \left\Vert y_n - {\hat{y}}\right\Vert _{Y}\left\Vert y_n + {\hat{y}}\right\Vert _{L^4(\Omega )}\left\Vert z\right\Vert _{L^4(\Omega )} \ dt\\&\quad \le C_4 \left\Vert y_n - {\hat{y}}\right\Vert _{L^2(0,T;Y)} \left[ \left\Vert y_n\right\Vert _{L^2(0,T;V)} + \left\Vert {\hat{y}}\right\Vert _{L^2(0,T;V)}\right] \left\Vert z\right\Vert _{L^{\infty }(0,T;V)}. \end{aligned}$$

Since *V* is compactly embedded in *Y*, we obtain by the Aubin Lions lemma that $\displaystyle \left\Vert y_n - {\hat{y}}\right\Vert _{L^2(0,T;Y)} \rightarrow 0$ for $n \rightarrow \infty$. This implies

$$\begin{aligned} \int _{0}^T \langle {{\mathcal {F}}}(y_n) - {{\mathcal {F}}}({\hat{y}}), z \rangle _{V^*,V} dt \xrightarrow [n \rightarrow \infty ]{} 0, \end{aligned}$$

and (A4) follows. It is simple to check that $\displaystyle {{\mathcal {F}}}'({\bar{y}}) = 2{\bar{y}} \in {{\mathcal {L}}}(L^2(I;V),L^2(I;V^*))$ and thus (A5) holds as well.

We turn to the assumption ${{\mathcal {F}}}({\bar{y}})\in C([0,T_{y_0});Y)$, for $y_0 \in {{\mathcal {D}}}({{\mathcal {A}}})$ and some $T_{y_0}$, arising in Theorem 3.2 for $y_0 \in {{\mathcal {D}}}({{\mathcal {A}}})$. Utilizing the fact that *V* embeds continuously into $L^4(\Omega )$ in dimension $d\le 4$ and ${\bar{y}} \in L^2(I;V)$, we have ${{\mathcal {F}}}(\bar{y}) \in L^2(I;Y)$. Hence parabolic regularity theory implies that ${\bar{y}} \in C([0,\infty );V)$ for $y_0 \in V$, and ${{\mathcal {F}}}({\bar{y}})\in C([0,\infty );Y)$ follows.

#### Remark 6.1

The specificity of this example rests in the fact that the second derivative is independent of the point were it is taken. Other nontrivial cases of analogous structure are reaction diffusion systems with bilinear coupling, see [13] where the finite horizon case was treated. Even the case of the Navier Stokes equations falls in this category. Sensitivity for the infinite horizon problems was treated by independent techniques in [6].

### Nonlinearities Induced by Functions with Globally Lipschitz Continuous Second Derivative

Consider the system (${\mathcal {P}}$) with ${{\mathcal {A}}}$ associated to a strongly elliptic second order operator with domain $H^2(\Omega ) \cap H^1_0(\Omega )$, so that (A1)–(A2) are satisfied. Let ${{\mathcal {F}}}: W_{\infty } \rightarrow L(I;V^*)$ be the Nemytskii operator associated to a mapping ${\mathfrak {f}}: {\mathbb {R}}\rightarrow {\mathbb {R}}$ which is assumed to be $C^2({\mathbb {R}})$ with first and second derivatives globally Lipschitz continuous, and second derivative globally bounded. The regularity assumption ${{\mathcal {F}}}({\bar{y}})\in C([0,T_{y_0});Y)$ for $y_0 \in V=H^1_0(\Omega )$ is satisfied by parabolic regularity theory. We discuss assumption (A3)–(A5) for such an ${{\mathcal {F}}}$, and show that they are satisfied for dimensions $d\in \{1,2\}$. For the finite horizon problem it will turn out that $d=3$ is also admissible. By direct calculation it can be checked that ${{\mathcal {F}}}$ is continuously Fréchet differentiable for $d \in \{ 1,2,3 \}$. We leave this part to the reader and immediately turn to the second derivative.

We proceed by considering the general dimension *d* to highlight, how the restrictions on the dimension arise. Thus let $d \in {\mathbb {N}}$ with $d>1$. The case $d=1$ can be treated with minor modifications from those in the following steps.

#### Second Derivative of ${{\mathcal {F}}}(y)$

For $y, h_1, h_2 \in W_\infty$ the relevant expression is given by

$$\begin{aligned}&\left\Vert {{\mathcal {F}}}'(y + h_2)h_1 - {{\mathcal {F}}}'(y)h_1 - {{\mathcal {F}}}''(y)(h_1,h_2)\right\Vert _{L^2(I;V^*)}\\&\quad = \sup _{\left\Vert \varphi \right\Vert _{L^2(I;V)} \le 1}\langle {{\mathcal {F}}}'(y + h_2)h_1 - {{\mathcal {F}}}'(y)h_1 - {{\mathcal {F}}}''(y)(h_1,h_2), \varphi \rangle _{L^2(I;V^*),L^2(I;V)}\\&\quad = \sup _{\left\Vert \varphi \right\Vert _{L^2(I;V)} \le 1} \int _{0}^{\infty } \int _{\Omega } ({\mathfrak {f}}'(y(t,x) + h_2(t,x))\\&\qquad - {\mathfrak {f}}'(y(t,x)) - {\mathfrak {f}}''(y(t,x))h_2(t,x)) h_1(t,x)\varphi (t,x) dxdt \\&\quad = \sup _{\left\Vert \varphi \right\Vert _{L^2(I;V)} \le 1} \int _{0}^{\infty } \int _{\Omega } g(t,x) \ h_2(t,x)h_1(t,x)\varphi (t,x) dxdt, \end{aligned}$$

where $$\displaystyle g(t,x) = \int _0^1 ({\mathfrak {f}}''(y(t,x) + sh_2(t,x)) - {\mathfrak {f}}''(y(t,x)))ds$$.

Note that *g* is bounded on $I \times \Omega$ and $g \in W_{\infty }$. Here we use that ${\mathfrak {f}}''$ is globally Lipschitz continuous and that $h_1 \in W_\infty$. Henceforth we let $r\in (1, \frac{2d}{d-2}]$ so that $W^{1,2}(\Omega )\subset L^r(\Omega )$ continuously. Let $r'$ denote the conjugate of *r* so that $r'\in [\frac{2d}{d+2} ,\infty )$ for $d>2$ and $r'\in (1,\infty )$ for $d=2$. We further choose $\rho> 1, \sigma > 2$ such that $\frac{1}{\rho } + \frac{2}{\sigma } = 1$. Then we estimate

$$\begin{aligned}&\left|\int _{0}^{\infty } \int _{\Omega } gh_1h_2\varphi \ dxdt\right|\\&\quad \le \int ^\infty _0 \left( \int _\Omega \left|g h_1 h_2\right|^{r'} dx \right) ^{{}^{1}\!/_{r'}} \left\Vert \varphi (t)\right\Vert _{L^r(\Omega )} \, dt \\&\quad \le \left( \int ^\infty _0 \left( \int _\Omega \left|g h_1 h_2\right|^{r'} dx \right) ^{{}^{2}\!/_{r'}}dt \right) ^{{}^{1}\!/_{2}} \left( \int ^{\infty }_0\left\Vert \varphi \right\Vert ^2_{L^r(\Omega )}dt \right) ^{{}^{1}\!/_{2}}. \end{aligned}$$

This further implies that

6.3 $$\begin{aligned}&\left|\int _{0}^{\infty } \int _{\Omega } gh_1h_2\varphi \ dxdt\right|\nonumber \\&\quad \le C_0 \left[ \int _{0}^{\infty } \left\Vert g(t)\right\Vert ^2_{L^{r'\rho }(\Omega )} \left\Vert h_1(t)\right\Vert ^2_{L^{r'\sigma }(\Omega )} \left\Vert h_2(t)\right\Vert ^2_{L^{r'\sigma }(\Omega )} dt\right] ^{{}^{1}\!/_{2}} \left\Vert \varphi \right\Vert _{L^2(I;V)} \longleftarrow (a). \end{aligned}$$

Here and below $C_i, i=0,1,2, \dots$ denote constant which are independent of $y,\varphi , h_1, h_2$. We next recall Gagliardo’s inequality [3, p 173]:

$$\begin{aligned} \left\Vert u\right\Vert _{L^q(\Omega )} \le \left\Vert u\right\Vert ^{1 - {}^{d}\!/_{q}-{}^{d}\!/_{2}}_{L^2(\Omega )}\left\Vert u\right\Vert ^{{}^{d}\!/_{2}+{}^{2}\!/_{q}}_{W^{1,2}(\Omega )}, \text { for all } q > 2, \text { and } u \in W^{1,2}(\Omega ) \equiv V, \end{aligned}$$

where $$q\in [2,2^*]$$ and

$$\begin{aligned} q^* {\left\{ \begin{array}{ll} \in [2,\infty ] \text { for } d = 1, \\ \in [2,\infty ) \text { for } d = 2, \\ \in [2, \frac{2d}{d-2}], \text { for } d > 2. \end{array}\right. } \end{aligned}$$

In the above estimate we take, $q = r'\sigma$. We obtain

$$\begin{aligned} 1 + \frac{d}{q} - \frac{d}{2}= & {} \frac{(2-d)r'\sigma + 2d}{2r'\sigma }, \ \frac{d}{2} - \frac{d}{q} \\= & {} \frac{d(r'\sigma - 2)}{2r'\sigma }, \text { also } r'\sigma> 2, \ (2-d)r'\sigma + 2d > 0. \end{aligned}$$

We estimate (6.3), (and check the conditions on the ranges of the parameters below)

$$\begin{aligned}&\sup _{\left\Vert \varphi \right\Vert _{L^2(I;V)} \le 1} (a)\\&\quad = C_1 \left( \int _{0}^{\infty } \left\Vert g(t)\right\Vert ^2_{L^{r'\rho }(\Omega )} \left( \left\Vert h_1(t)\right\Vert _{L^2(\Omega )} \left\Vert h_2(t)\right\Vert _{L^2(\Omega )} \right) ^{\frac{(2-d)r'\sigma + 2d}{r'\sigma }} \right. \\&\left. \qquad \left( \left\Vert h_1(t)\right\Vert _{V} \left\Vert h_2(t)\right\Vert _{V} \right) ^{\frac{d(r'\sigma - 2)}{r'\sigma }} \right) ^{{}^{1}\!/_{2}}dt \\&\quad \le C_2 \left( \left\Vert h_1\right\Vert _{W_{\infty }} \left\Vert h_2\right\Vert _{W_{\infty }} \right) ^{\frac{(2-d)r'\sigma + 2d}{2r'\sigma }} \\&\qquad \left( \int _{0}^{\infty } \left\Vert g(t)\right\Vert ^2_{L^{r'\rho }(\Omega )} \left( \left\Vert h_1(t)\right\Vert _{V} \left\Vert h_2(t)\right\Vert _{V} \right) ^{\frac{d(r'\sigma - 2)}{r'\sigma }} \right) ^{{}^{1}\!/_{2}}dt \longleftarrow (b) \end{aligned}$$

We set $\frac{d(r'\sigma - 2)}{r'\sigma } = \frac{2}{3}$. This yields,

6.4 $$\begin{aligned} (b)&\le C_3 \left( \left\Vert h_1\right\Vert _{W_{\infty }} \left\Vert h_2\right\Vert _{W_{\infty }} \right) ^{{}^{2}\!/_{3}} \left( \left\Vert h_1\right\Vert _{W_{\infty }} \left\Vert h_2\right\Vert _{W_{\infty }} \right) ^{{}^{1}\!/_{3}} \left( \int _{0}^{\infty } \left\Vert g(t)\right\Vert ^6_{L^{r'\rho }(\Omega )}\right) ^{{}^{1}\!/_{6}} dt \nonumber \\&= C_4 \left\Vert h_1\right\Vert _{W_{\infty }} \left\Vert h_2\right\Vert _{W_{\infty }} \left( \int _{0}^{\infty } \left( \int _{\Omega } \left|g(t)\right|^{r'\rho } dx \right) ^{{}^{6}\!/_{r'\rho }} dt \right) ^{{}^{1}\!/_{6}} . \end{aligned}$$

Now we check the conditions on the parameter $r, \sigma , d$ , and $r', \sigma$. Since $\frac{d(r'\sigma - 2)}{r'\sigma } = \frac{2}{3}$, together with the conditions on $r'$ and $\sigma$ these parameters need to satisfy

6.5 $$\begin{aligned} r'\sigma = \frac{6d}{3d-2}, \quad r'\in \left[ \frac{2d}{d+2}, \infty \right) , \quad \sigma \in (2,\infty )\quad r'\sigma \in [2,2^*] , \end{aligned}$$

and $r'>1$ if $d=2$. The last condition above holds without restricting the dimension *d*. From the first three relations we infer that necessarily $\frac{6d}{3d-2} =r'\sigma >\frac{4d}{d+2}$ which is only possible for $d\le 3$.

*Let us focus on* $d=2$. Then the choice of parameters $r=6,r'={}^{6}\!/_{5}, \sigma ={}^{5}\!/_{2}, \rho =5$ satisfies all the above requirements and it is convenient to further estimate (6.4). In fact we obtain

$$\begin{aligned}&\left\Vert {{\mathcal {F}}}'(y + h_2)h_1 - {{\mathcal {F}}}'(y)h_1 - {{\mathcal {F}}}''(y)(h_1,h_2)\right\Vert _{L^2(I;V^*)} \\&\quad \le C_5 \left\Vert h_1\right\Vert _{W_{\infty }} \left\Vert h_2\right\Vert _{W_{\infty }} \left( \int _{0}^{\infty } \left( \int _{\Omega } \left|g(t,x)\right|^{6}dx \right) dt \right) ^{{}^{1}\!/_{6}}\\&\quad \le C_6 \left\Vert h_1\right\Vert _{W_{\infty }} \left\Vert h_2\right\Vert _{W_{\infty }} \left( \int _{0}^{\infty } \left( \int _{\Omega } \left|g(t,x)\right|^{2}dx \right) dt \right) ^{{}^{1}\!/_{6}} \end{aligned}$$

for all $y, h_1, h_2 \in W_\infty$. Here we use the boundedness of *g*. By Lebesgue’s bounded convergence theorem the last factor converges to 0 for $\left\Vert h_2\right\Vert _{W_\infty } \rightarrow 0$ and hence the fact that ${{\mathcal {F}}}$ is twice differentiable is verified. The continuity of the second derivative follows with the above estimates and again by the Lebesgue theorem.

*Next we consider* $d=3$. In this case an analogous procedure is not possible, since the relations (6.5) and $r'\rho \le 6$ cannot be fulfilled simultaneously. In fact, $r'\sigma = {}^{18}\!/_{7}, r'\ge {}^{6}\!/_{5}$, and thus necessarily $\sigma \in (2, {}^{15}\!/_{7}]$. The condition $r'\rho \le 6$ is equivalent to $12 \le \sigma (6-r')={}^{18}\!/_{7r'}(6-r')$, which in turn is equivalent to $17r' \le 18$, which contradicts $r' \ge {}^{6}\!/_{5}$.

Thus we fix parameters *r* and $\sigma$ such that (6.5) are satisfied for $d=3$, as for instance $r'={}^{6}\!/_{5}, \sigma = {}^{15}\!/_{7}$, which implies that $\rho =15$ and $r'\rho =18$. Then for the *finite horizon problem* we can estimate by Hölder’s inequality with $\eta = {}^{r'\rho }\!/_{6}$:

$$\begin{aligned} (b)&\le C_7 \left\Vert h_1\right\Vert _{W_{\infty }} \left\Vert h_2\right\Vert _{W_{\infty }} \left( \int _{0}^{T} \left( \int _{\Omega } \left|g(t,x)\right|^2 dx \right) ^{{}^{6}\!/_{r'\rho }}dt \right) ^{{}^{1}\!/_{6}} \nonumber \\&\le C_8 \left\Vert h_1\right\Vert _{W_{\infty }} \left\Vert h_2\right\Vert _{W_{\infty }} \left( \int _{0}^{T} \left( \int _{\Omega } \left|g(t,x)\right|^2 dx \right) dt\right) ^{{}^{1}\!/_{6\eta }} T^{{}^{1}\!/_{6\eta '}}. \end{aligned}$$

From here we can proceed as in the case $d=2$ to assert the continuous second Fréchet differentiability of ${{\mathcal {F}}}$ in $d=3$ for the finite horizon case.

#### Assumptions (A4) and (A5)

In order to verify (A4), we show that ${{\mathcal {F}}}(y):W(0,T) \rightarrow L^1(0,T;V^*)$ is continuous for every $T > 0$. We consider the sequence $y_n \rightharpoonup {\hat{y}}$ in *W*(0, *T*) and let $z \in L^{\infty }(0,T;V)$ be given. Then we estimate

$$\begin{aligned} \int _{0}^T \langle {{\mathcal {F}}}(y_n) - {{\mathcal {F}}}({\hat{y}}), z \rangle _{V,V^*} dt= & {} \int _{0}^T \int _{\Omega } ({\mathfrak {f}}(y_n)\\&- {\mathfrak {f}}({\hat{y}}))z \ dxdt \le C \left\Vert y_n - {\hat{y}}\right\Vert _{L^2(0,T;Y)}\left\Vert z\right\Vert _{L^2(0,T;Y)}. \end{aligned}$$

Then by the compactness of *V* in *Y*, we obtain (A4).

Now we verify (A5). We recall Remark 3.1, and proceed as in (6.2) for $y \in W_{\infty }, \varphi \in L^2(I;V)$,

$$\begin{aligned}&\left\Vert {{\mathcal {F}}}'(y)^*p\right\Vert _{L^2(I;V^*)} \\&\quad = \left\Vert ({{\mathcal {F}}}'(y)^* - {{\mathcal {F}}}'(0)^*)p\right\Vert _{L^2(I;V^*)}\\&\quad = \sup _{\left\Vert \varphi \right\Vert _{L^2(I;V)} \le 1} \int _{0}^{\infty }\int _{\Omega } \langle ({{\mathcal {F}}}'(y)^* - {{\mathcal {F}}}'(0)^*)p, \varphi \rangle _{V^*,V} \\&\quad = \sup _{\left\Vert \varphi \right\Vert _{L^2(I;V)} \le 1} \int _{0}^{\infty }\int _{\Omega } ({\mathfrak {f}}'(y) - {\mathfrak {f}}'(0))p\varphi \ dxdt \le C\left\Vert y\right\Vert _{W_{\infty }}\left\Vert p\right\Vert _{L^2(I;V)}. \end{aligned}$$

This shows ${{\mathcal {F}}}'(y)^*$ satisfies (A5).

### Cubic Nonlinearity $y^3$ in One Dimension ($\Omega \subset {\mathbb {R}}$)

We can also consider the optimal stabilization problem with cubic nonlinearity, i.e. ${{\mathcal {F}}}(y) = y^3$ in one dimension. This is a special monotone case of the Schlögl model of theoretical chemistry.

6.6a $$\begin{aligned} ({\mathcal {P}}_{Sch}) \qquad {\mathcal {V}}(y_0) = \min _{\begin{matrix} (y,u) \in W_\infty \times U_{ad} \end{matrix}} \ \frac{1}{2} \int _{0}^{\infty } \left\Vert y\right\Vert ^2_Y dt + \frac{\alpha }{2} \int _{0}^{\infty } \left\Vert u\right\Vert ^2_{{{\mathcal {U}}}} dt, \end{aligned}$$

subject to 
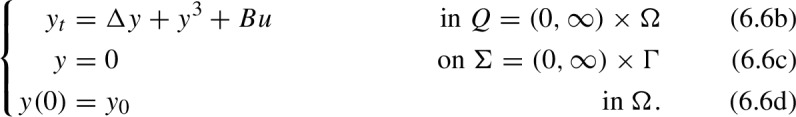
 In this model, one can easily verify assumption (A1) is satisfied by taking ${{\mathcal {A}}}y= \Delta y, \ y|_{\Gamma } = 0,$ and ${{\mathcal {D}}}({{\mathcal {A}}}) = H^2(\Omega ) \cap V$. Clearly ${{\mathcal {A}}}$ can be extended to ${{\mathcal {A}}}\in {{\mathcal {L}}}(V,V^*)$. Moreover ${{\mathcal {A}}}$ generates an analytic semigroup on *Y* which is uniformly stable. Assumption (A2) is satisfied under the same argumentation as in Fisher’s equation. Differentiability assumption (A3), and continuity assumption (A4) are satisfied along similar computations as in Sects. 6.2.1, 6.2.2. For (A5) we require that $y_0\in V$. Indeed in this case for ${\bar{y}} \in W_\infty$ by Gagliardo’s inequality

$$\begin{aligned} \int _0^\infty \int _\Omega |{\bar{y}}^3|^2 dx dt= & {} \int _0^\infty \left\Vert {\bar{y}}\right\Vert ^6_{L^6(\Omega )} dt \le \int ^\infty _0\left\Vert \bar{y}\right\Vert ^4_{L^2(\Omega )} \left\Vert {\bar{y}}\right\Vert ^2_{V} dt\\\le & {} C \left\Vert \bar{y}\right\Vert ^4_{W_\infty }\int ^\infty _0 \left\Vert {\bar{y}}\right\Vert ^2_{V} dt \le C \left\Vert {\bar{y}}\right\Vert ^6_{W_\infty }. \end{aligned}$$

Thus ${\bar{y}}^3 \in L^2(I;Y)$ and parabolic regularity theory implies that ${\bar{y}}\in C(I;V)$ if $y_0\in V$. We estimate for $h,\varphi \in L^2(I;V)$, suppressing the arguments (*t*, *x*),

$$\begin{aligned}&\left|\int _0^\infty \int _{\Omega } {{\mathcal {F}}}'({\bar{y}}) h \varphi \, dx dt\right| \le \left|\int _0^\infty \int _{\Omega } {\bar{y}}^2 h \varphi \, dx dt\right| \\&\quad \le \int _0^\infty \left\Vert {\bar{y}}\right\Vert ^2_{L^4(\Omega )} \left\Vert h\right\Vert _{L^4(\Omega )} \left\Vert \varphi \right\Vert _{L^4(\Omega )} \, dt, \\&\quad \le C \left\Vert {\bar{y}}\right\Vert ^2_{C(I;V)} \left\Vert h\right\Vert _{L^2(I;V)} \left\Vert \varphi \right\Vert _{L^2(I;V)} \end{aligned}$$

which implies (A5). Moreover we have ${{\mathcal {F}}}(\bar{y})\in C([0,T_{y_0});Y)$, since $V \subset C({{\bar{\Omega }}})$ in dimension 1, and thus the extra regularity demanded in Theorem 3.2 is satisfied.
